# Forensic Geochemistry Reveals International Ship Dumping as a Source of New Oil Spill in Brazil’s Coastline (Bahia) in Late 2023

**DOI:** 10.1021/acs.est.4c01520

**Published:** 2024-05-13

**Authors:** Laercio L. Martins, Vinícius B. Pereira, Adriana P. Nascimento, Rufino Neto A. Azevedo, André H. B. Oliveira, Carlos Eduardo P. Teixeira, Débora A. Azevedo, Georgiana F. da Cruz, Rivelino M. Cavalcante, Tommaso Giarrizzo

**Affiliations:** † Laboratory of Petroleum Engineering and Exploration (LENEP), North Fluminense State University (UENF), Macaé 27925-535, Rio de Janeiro, Brazil; ‡ Institute of Marine Sciences (LABOMAR), Federal University of Ceará (UFC), Fortaleza 60165-181, Ceará, Brazil; § Institute of Chemistry (IQ), Federal University of Rio de Janeiro (UFRJ), Rio de Janeiro 21941-598, Brazil; ∥ Chemistry and Physical Chemistry Department (DQAFQ), Federal University of Ceará (UFC), Fortaleza 60455-760, Ceará, Brazil

**Keywords:** oil dumping, tarballs, international waste, marine pollution, northeastern Brazil, environmental geochemistry

## Abstract

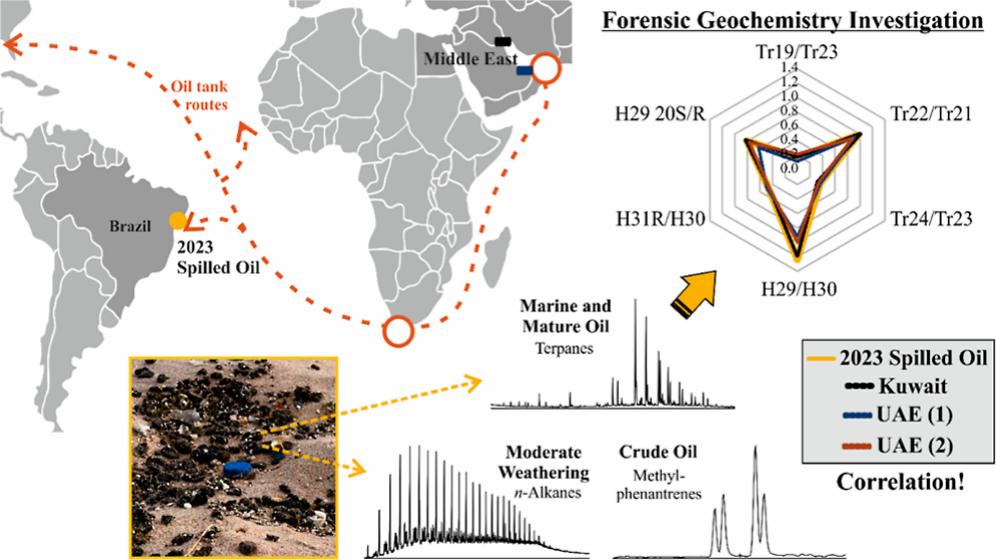

In the present study, we applied forensic geochemistry to investigate the origin and fate of spilled oils like tarballs stranded at the beaches of Bahia, in northeastern Brazil, in September 2023, based on their fingerprints. Saturated and aromatic compounds were assessed by gas chromatography, and the oceanic surface circulation patterns were deciphered to determine the geographic origin of the spill. Contamination by petroleum represents an enormous threat to the unique, species-rich ecosystems of the study area. The geochemical fingerprint of the oil spilled in 2023 did not correlate with those of previous events, including the one in 2019, the one in early 2022 in Ceará, and an extensive spill across the Brazilian Northeast in late 2022. However, the fingerprint did correlate with crude oils produced by Middle Eastern countries, most likely Kuwait. The oil of the 2023 spill had a carbonate marine origin from early mature source rocks. These findings, together with the moderate weathering of the 2023 tarballs and the ocean circulation patterns at the time of the event, indicate that the oil was discharged close to the shore of Brazil, to the east or southeast of Salvador, by a tanker on an international route in the South Atlantic.

## Abstract 2

Orphan oil spills contaminate the Brazilian coast regularly. This study attributes the latest event to the dumping of crude oil of Middle Eastern origin by tankers on South Atlantic routes.

## Introduction

In September 2023, an oil spill from an unknown source reached the coast of Bahia, in northeastern Brazil (the oil spill contextualization is presented in the Supporting Information), contaminating many local beaches and coastal areas.^1−4^ The first tarballs were found on the beaches of the state capital, Salvador. Over the following few days, these contaminants were found further south, along approximately 120 km of the coast of Bahia.^5^ This coast is an area of unique ecological significance, which encompasses estuarine and coral ecosystems with high species richness and endemism, including the “Baía de Todos os Santos” Environmental Protection Area, in addition to the marine habitats further south on the Abrolhos Banks, the richest coral reef complex in the Southwest Atlantic.^6−8^

In recent decades, oil pollution has become a persistent environmental and socioeconomic concern on a global scale.^9^ Oil spills have now become a chronic issue on the coast of northeastern Brazil, with countless social, economic, and ecological impacts, given the contamination of many protected areas and coastal communities.^7,10^ In 2019, the coast of northeastern Brazil was hit by the largest oil spill ever recorded in the tropical ocean,^11^ with more than 5000 tons of oil being recovered from the beaches of 11 states.^7^ Contaminated materials related to this event still reached Brazilian beaches in 2020 and 2021.^12^

In January 2022, a novel oil spill unrelated to the 2019 event was recorded on the coast of Ceará, which affected 400 km of the shore with more than 8000 L of oil.^13,14^ In late 2022, tarballs were washed up all along the coast of northeastern Brazil.^15−17^ In most cases, the oil has not been produced in Brazil but has apparently been dumped in international waters by passing ships before being carried ashore by ocean currents, representing a worldwide issue.^18,19^

Forensic environmental geochemistry has been applied successfully to the assessment of petroleum-related contaminants in marine environments. This approach can determine the source of these events, the timing of their release, and the weathering processes that affect the contaminants based on the scientific principles of organic geochemistry.^20^ The analytical techniques employed here, in particular, conventional gas chromatography (GC), can be used to identify molecular biomarkers and, in turn, define the chemical fingerprint of the contaminants.^21,22^ The methodology typically involves the assessment of the most significant factors that determine the chemical fingerprint of the oil spill, including its origin, genesis, refinement, weathering processes, and environmental mixing.^21^ This approach has been employed amply to examine and diagnose the oil spills that have affected the coast of northeastern Brazil in recent years.^11−13,16,17,23−28^

In this context, the present study assesses the origin and characteristics of the oil that washed up on the coast of the state of Bahia, in northeastern Brazil, in late 2023, highlighting the multiple sources of these recurrent events. To this end, forensic environment geochemistry was applied to determine (i) the possible similarities between the new material and previous oil spills, (ii) the origin of these contaminants, (iii) their geochemical characteristics (genesis), (iv) whether the material is crude or fuel oil, and (v) its level of weathering. Ocean circulation patterns during the period of the event were also assessed to determine the possible route of the oil prior to its arrival on the Brazilian coast and the location of its dumping. The study also intends to highlight the inadequate monitoring of the dumping of oil by ships on the international routes of the South Atlantic Ocean and encourage more effective regulations and international cooperation for the prevention of oil pollution.

## Materials and Methods

### Sample Set

**Figure 1:**
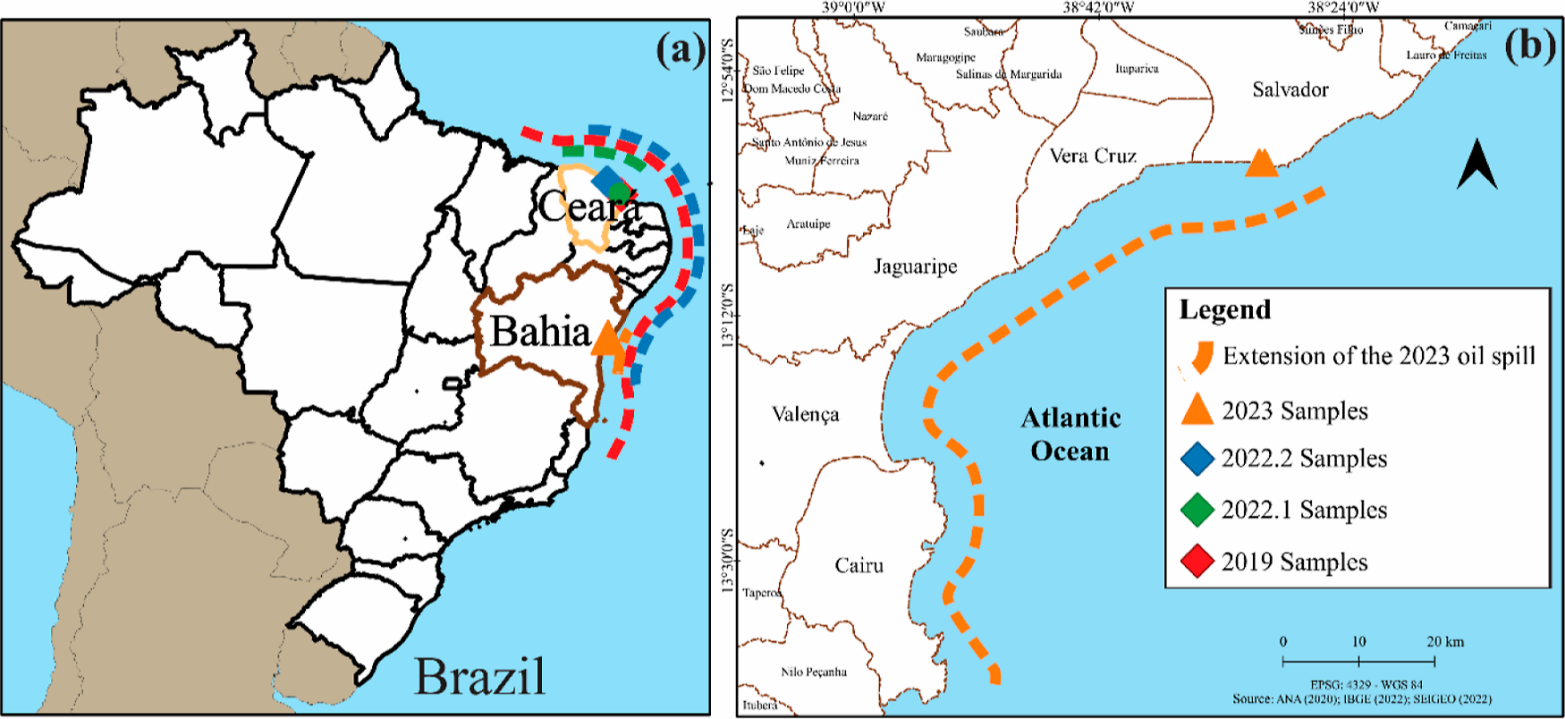
(a) Locations of the samples obtained from the 2022.2, 2022.1, and 2019 oil spills, which were included in the analyses, and the extension of each event represented by the blue, green, and red dotted lines, respectively. (b) Location of the oil samples collected from Ondina and Paciência beaches in the city of Salvador, state of Bahia, northeastern Brazil, in September 2023. The orange dotted line indicates the extension of the coastline affected by the 2023 oil spill. Further details are provided in Table S1.

**Figure 2:**
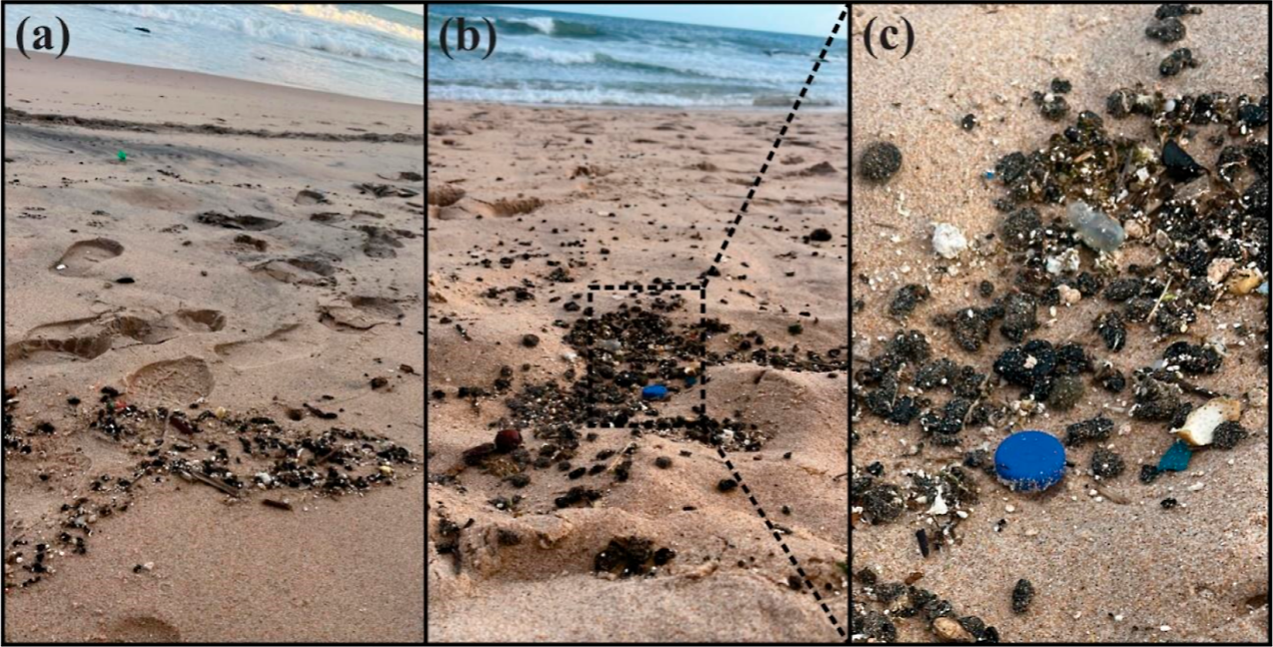
Tarballs found on (a) Ondina beach and (b,c) Paciência beach, on the coast of Salvador, capital of Bahia state, in northeastern Brazil, in September 2023.

For analysis of this event, four oil samples (PP01#2023, PP02#2023, PO01#2023, and PO02#2023) were collected on September 11, 2023, from Paciência and Ondina beaches, in the city of Salvador, capital of Bahia state, in northeastern Brazil (Figure 1), as soon as they were stranded at the seashore. The oil-like tarballs were taken from the high tide line that was almost completely covered with oily material over the entire length of the affected beaches (Figure 2). The analysis also included two samples from the 2019 oil spill (P01#2019 and P03#2019),^11^ two samples from the oil spill of early 2022 on the coast of Ceará (P04#2022.1 and P05#2022.1),^13^ and two samples of the tarballs (P01#2022.2 and P02#2022.2)^16^ that beached on the coast of Brazil in late 2022 (details in Table S1).

The 2023 oil spill comprised small tarballs ranging in diameter up to 3 cm (Figure 2). These tarballs were soft, black, and viscous, with the general physical appearance of petroleum, and were floating on the surface of the water.^3^ The total amount of stranded oil in 2023 was lower than that in the previous oil spill events but still enough to affect at least 16 beaches in five coastal cities, spanning approximately 120 km of coastline. The 2023 samples have some physical similarities with the previous beached oily materials in 2019, 2022.1, and 2022.2, presenting a dark color, strong odor, and tar characteristics (Figure 2). However, while the spilled oil in 2019 was a viscous liquid,^27,28^ the 2022.1 spilled oil was small fragments and viscous liquid oils,^13,14^ and the 2022.2 spill consisted of small to thick tarballs.^16,17^

### Oil Extraction and Separation

Approximately 1–2 g of each tarball mixed with sediments was extracted with a solution of dichloromethane or dichromethane/methanol.^24,29^ The asphaltene was precipitated,^30,31^ and the maltene was fractionated into the saturated, aromatic, and resin components.^30,32^ A detailed description of this process is presented in the Supporting Information.

### Gas Chromatography-Flame Ionization Detector

The analyses of the saturated hydrocarbon fractions of the 10 samples were conducted on an Agilent 7890A gas chromatograph (Agilent Technologies, Palo Alto, CA, USA) equipped with an HP-5MS column (5% phenyl–95% dimethylpolysiloxane, 30 m × 0.25 mm i.d., 0.25 μm df). The chromatography method was as follows: injector at 300 °C in splitless mode, oven temperature program from 40 to 320 °C at 6 °C min^–1^, maintained for 10 min, and detector operating at 350 °C. Hydrogen (99.9990% pure) was used as the carrier gas at a constant flow of 1.7 mL min^–1^. The data were processed in the EZChrome Elite program. The target compounds were the *n*-alkane homologous series and the isoprenoid hydrocarbons pristane (Pr) and phytane (Ph).

### Gas Chromatography–Mass Spectrometry

The saturated and aromatic hydrocarbon fractions were analyzed using an Agilent Technologies 6890N gas chromatograph–mass spectrometer. The gas chromatograph was equipped with an HP-5MS (5% phenyl–95% methylsiloxane, 30 m, 0.25 mm i.d., 0.25 μm df; Agilent Technologies). The analytical conditions followed those described by Lima et al.^12^ The mass spectrometer operated in the scan (50–600 u) and selected ion monitoring modes. The target biomarkers were the saturated compounds tri-, tetra-, and pentacyclic terpanes (*m*/*z* 191), steranes (*m*/*z* 217, 218), C27 diasteranes, and C30 tetracyclic polyprenoids (TPPs, *m*/*z* 259) and the aromatic compounds regular benzohopanes (*m*/*z* 191), triaromatic steranes (TAS; *m*/*z* 231), and the monoaromatic 8,14-secohopanes (*m*/*z* 365). The target polycyclic aromatic hydrocarbons (PAHs) were the parental and the homologous methylated series of the naphthalenes (*m*/*z* 128, 142, 156, 170, and 184), fluorenes (*m*/*z* 166, 180, 194, and 208), phenanthrenes (*m*/*z* 178, 192, 206, 220, and 234), and chrysenes (*m*/*z* 228, 242, and 256), in addition to the sulfur aromatic compound dibenzothiophene and its methylated series (*m*/*z* 184, 198, 212, and 226). Semiquantification of the aromatic compounds was obtained by internal standardization, using pyrene-*d*_10_ at a 5.9 μg mL^–1^ concentration (for more details, see Lima et al.^12^).

### Multivariate Statistical Analysis

A shade plot combined with a cluster analysis using the Euclidean distance was used to assess the similarities in the diagnostic ratios among the oil samples collected from the northeastern coast of Brazil between 2019 and 2023. This included two samples from each of the three previous oil spills (2019, 2022.1, and 2022.2), along with four samples from 2023 (Table S1). The same approach was employed to assess the similarities between the four 2023 samples and 20 crude oils of different petroleum exporting countries. The similarity profile test (SIMPROF, *p* < 0.005) was applied to determine the significance of the differences between the clusters.^33^ This analysis was run with 9999 permutations to test the null hypothesis that there was no meaningful group structure. The multivariate analyses were run in PRIMER 7 with the PERMANOVA add-on.^34^

### Ocean Circulation

Daily data on the mean surface currents of the Atlantic Ocean were obtained for the period between September 10 and 15th, 2023, from the Global Ocean Reanalysis and Simulation (GLORYS-MERCATOR) project, run PHY_001_024. These data were used to characterize the ocean circulation in the study area during the period of the oil spill event. These data are based on hourly outputs and include the residual effects of the local tides and the Stokes drift from the waves. The GLORYS-MERCATOR project has already been validated for the study region with data from the ocean surface current analysis real-time project. It has also been compared to the ADCP current measurements at Forte beach, on the Bahia coast north of Salvador (12.6°S) at a depth of 32 m.^35^

## Results and Discussion

### Evaluation of the Similarities among the Northeast Brazilian Spilled Oils

**Figure 3:**
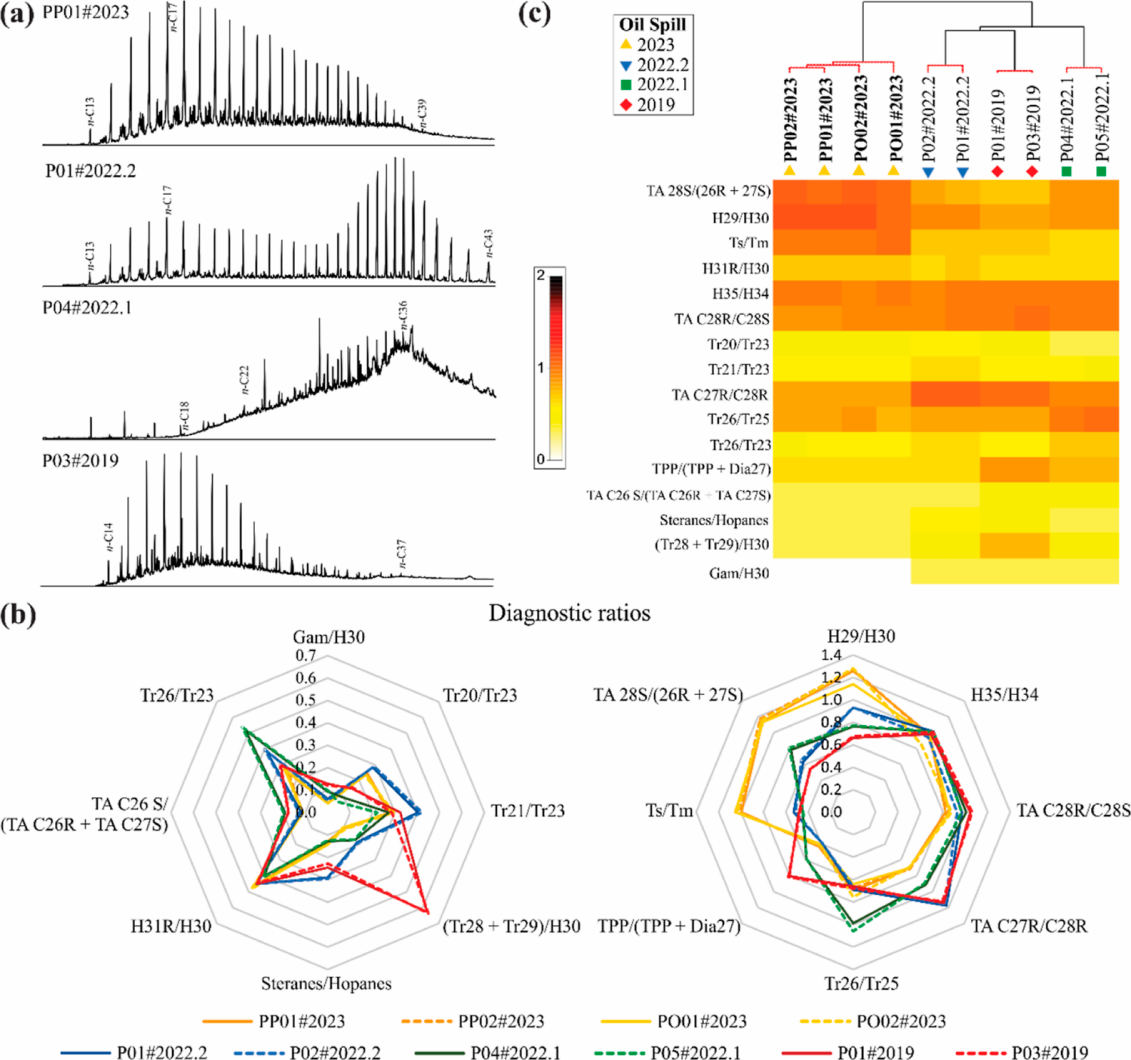
(a) Representative GC-FID chromatograms of the saturated fractions of the samples from each oil spill event. (b) Radar plots comparing the diagnostic ratios derived from the terpanes, steranes, triaromatic steroids, and tetracyclic polyprenoids of the four oil samples collected in 2023, the two oil samples collected in late 2022 (2022.2), the two tarball samples collected in early 2022 (2022.1), and the two oil samples collected in 2019. (c) Cluster analysis and shade plot of the diagnostic ratios of the 10 oil samples from the northeastern Brazilian coast collected between 2019 and 2023. The samples and nodes that did not vary significantly from one another (SIMPROF test) are connected by red lines.

The GC-FID chromatogram profiles are highly similar among the four 2023 samples (Figure S1a–d), with a bimodal shape, low- to medium unresolved complex mixture (UCM), and *n*-alkanes ranging from C13 to C39. However, they are quite distinct from the GC profiles of the previous samples (Figure 3a). In particular, while the 2022.2 samples are bimodal in shape, with a low UCM (Figure S1e,f),^16,17^ the *n*-alkanes of the 2022.1 samples are depleted significantly, with a very high UCM (Figure S1g,h),^13^ and the 2019 samples are unimodal, with a high UCM (Figure S1i,j).^12^ These findings were the initial evidence that the different sets of samples have distinct sources and that the 2023 oil spill represents a novel event off the Brazilian coast.

In addition, we investigated possible similarities between the 2023 oil spill and previous spills on the northeastern Brazilian coast using petroleum molecular biomarkers resistant to weathering processes.^36−38^ These biomarkers were analyzed by GC–MS and included the tricyclic and pentacyclic terpanes (monitoring *m*/*z* 191; selected ion chromatograms are shown in Figure S2), steranes (monitoring *m*/*z* 217; see Figure S3), triaromatic steroids (TAS; monitoring *m*/*z* 231; see Figure S4), and tetracyclic polyprenoids (TPPs; monitoring *m*/*z* 259; see Figure S5). We found some dissimilarities in the distribution of these biomarkers (Figure S2) when comparing the 2023 samples with those from 2019, early 2022 (2022.1), and late 2022 (2022.2), including a higher abundance of the C30 hopane (H30) in comparison with the C29 hopane (H29). The terpanes could be altered only under severe weathering conditions and over a long time of exposure,^36,39^ which is not the case for the 2023 samples that still have the most susceptible to biodegradation *n*-alkanes in high abundance. Additionally, their relative proportion, including H29 and H30, was shown not to change one and a half years after the 2019 oil spill in Brazil.^12^

The lack of similarity among these oil samples is best exemplified by the significant differences found among diagnostic ratios based on the resistant biomarkers (Table S2), as shown in the radar plots generated by EXCEL (Figure 3b). In general, these ratios are known to be very stable, even under the tropical conditions of the Brazilian coast^12,26^ and values of relative standard deviation (RSD) less than 10% can indicate similarities among spilled oils.^11^ The 16 ratios recorded here (Figure 3b) indicate that the four 2023 samples are highly similar to one another, with a RSD of less than 6.0% for all of them (Table S2; calculated by EXCEL). When the two samples from each of the other three previous oil spill events (2022.2, 2022.1, and 2019) were added to the analysis, however, the values of RSD for 15 diagnostic ratios were considerably higher than the values only for the 2023 samples (Table S2). In addition, at least half of the ratios presented values of RSD higher than 10%, including eight ratios considering the four 2023 and two 2022.2 samples (13.3–40.1%), 11 ratios considering the four 2023 and two 2022.1 samples (12.2–41.6), and 11 ratios considering the four 2023 and two 2019 samples (10.4–90.2%, Table S2). Therefore, these findings indicate that the 2023 samples did not correlate with any of the samples from previous years.

Furthermore, the SIMPROF test, combined with the cluster analysis, identified four significantly distinct groups of oil samples from the northeastern Brazilian coast (Figure 3c). In this analysis, the most distinct cluster was formed by the samples from the 2023 event, which was characterized by the highest TA 28S/(26R + 27S), H29/H30, and Ts/Tm values.

### Origin of the 2023 Oil Spill

The dumping of oil by ships on route across the South Atlantic Ocean is thought to be a frequent phenomenon.^18^ Oil tankers are allowed to do operational discharges of oil only following restricted conditions, such as when not in a special area, with more than 50 nautical miles distance from the land, and not exceeding 30 L per nautical mile, among others (details in Zacharias et al.^18^). However, many of them benefit from insufficient surveillance to illegally discharge oil in the ocean during operational activities, including cleaning bilges and tanks.^18^ Accidental discharge of oil is also related.

Oil has reached the Brazilian coast, in particular the equatorial northern and northeastern shores, on a regular basis.^18,40^ This conclusion is supported by a large number of oil spills of unrelated origin—invariably produced in foreign countries—that reach the Brazilian coast.^11,13,17,23,25,41^ In addition, the coastal regions most vulnerable to oil spills are adjacent to oil tanker routes.^9,40,42^ Given this, we compared the fingerprint of the 2023 samples with the oil profiles from the leading crude oil exporting countries in the Middle East (Saudi Arabia, Iran, Kuwait, United Arab Emirates, and Oman), Africa (Angola, Nigeria, Libya, Algeria, and Gabon), and Latin American (Brazil, Colombia, and Venezuela; Figure S6).^43,44^ The South Atlantic Ocean is part of the maritime transportation routes for the crude oil trade of these countries (Figure S6).^43,45^

**Figure 4:**
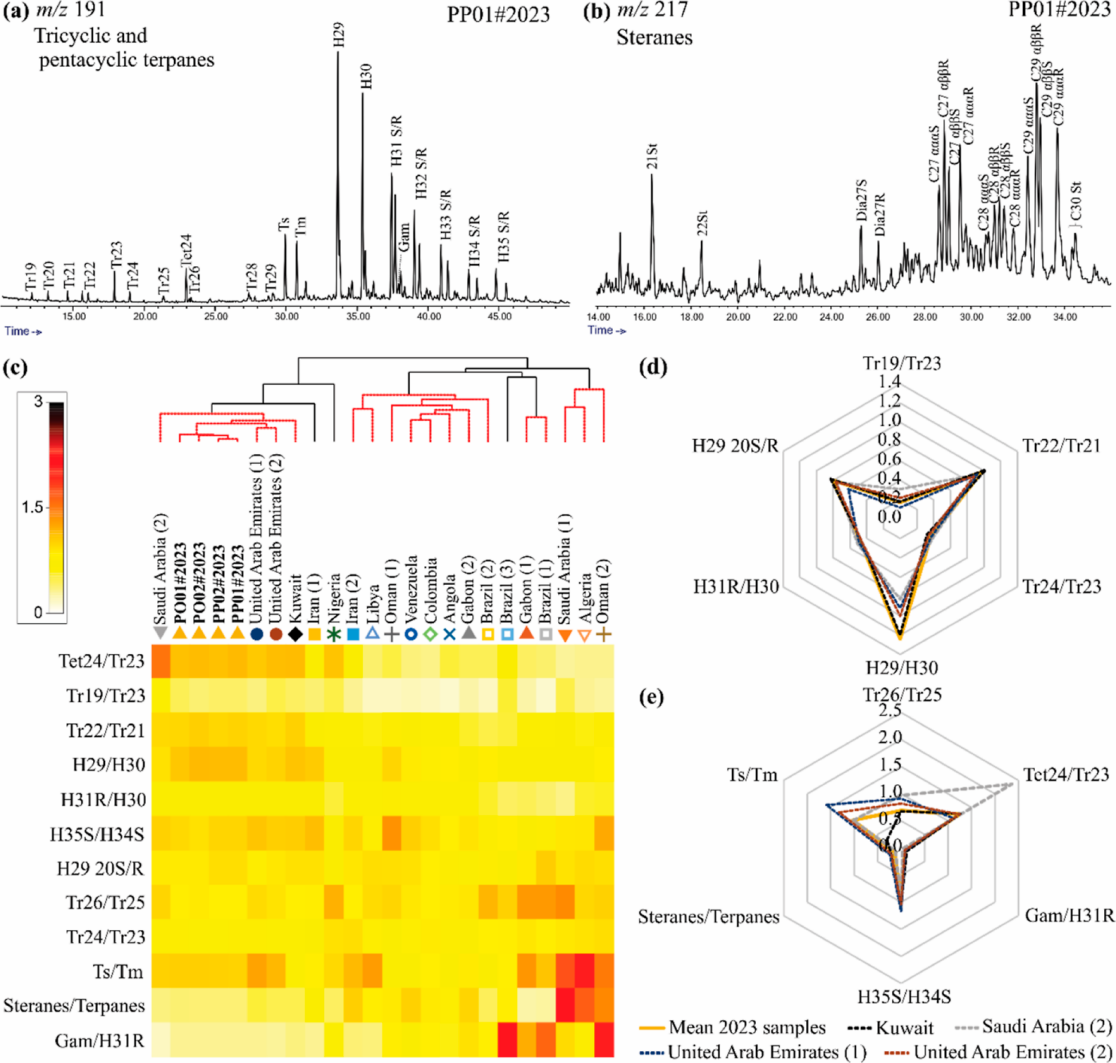
Mass chromatograms obtained from the GC–MS analysis, showing the distribution of the (a) terpanes (*m*/*z* 191) and (b) steranes (*m*/*z* 217). (c) Cluster analysis and shade plot of the diagnostic ratios of the terpanes and steranes of the 2023 samples, together with those of crude oil produced by countries in the Middle East (Saudi Arabia, Iran, Kuwait, United Arab Emirates, and Oman), Africa (Angola, Nigeria, Libya, Algeria, and Gabon), and South America (Brazil, Colombia, and Venezuela). The samples and nodes that did not vary significantly (SIMPROF test) are connected by red lines. (d,e) Radar plots comparing the diagnostic ratios between the 2023 samples (mean value for the four samples) and the most similar crude oils, that is, the samples from Kuwait, Saudi Arabia, and the United Arab Emirates (data from Peters et al.^46^).

We compared diagnostic ratios (Table S3) based on the recalcitrant petroleum biomarkers, tricyclic and pentacyclic terpanes and steranes of the samples, which can be visualized through the representative chromatograms presented in Figure 4a,b. We compared the ratios of the 2023 samples with those of crude oils from the leading crude oil exporting countries (Figure S6) using data available in Peters et al. (geological information on the crude oils in Table S4).^46^

The crude oils from Kuwait (Magwa field; Burgan Formation of the Burgan Rumaila Hight Basin), Saudi Arabia (Raghib field; Unayzah Formation of the Western Platform Basin), and the United Arab Emirates (Zakum and Upper Zakum fields; Shilaif and Thamama formations of the Southern Arabian Gulf) are the most similar to the 2023 sample, according to the cluster analysis, which links them significantly, based on the results of the SIMPROF test (Figure 4c). These Middle Eastern countries are responsible for a major proportion of the world’s oil trade.^47^

In addition, the diagnostic ratios of the 2023 samples are even more similar to those of the crude oil from Kuwait (Figures 4d,e and S7).^3^ For example, they have a higher H29/H30, Tr22/Tr21, Gam/H31R, and H29 S/R ratios values than the other oils, and lower Tr26/Tr25 (Figure 4d,e; Table S3). The Saudi Arabian (2) crude oil is the most distinct, with a much higher abundance of C24 tetracyclic terpanes (high Tet24/Tr23, Figure 4e) and lower C29 hopane (low H29/H30, Figure 4d), in comparison with the 2023 samples. The crude oils from the United Arab Emirates also have lower H29/H30 ratio values than the 2023 samples.

The Kuwaiti crude oil was produced from the Magwa field (Burgan Formation, lower to middle Albian) of the Burgan Rumaila Hight Basin. It was generated from the marine carbonate source rocks of the Jurassic Najmah Formation (details in Table S4).^46,48^ The Magwa field is part of the Greater Burgan field, the world’s second-largest oilfield, and the largest known clastic oilfield.^49−51^ Kuwait is also the seventh-largest oil-exporting country worldwide.^47^

To confirm the probable origin of the 2023 samples, their *m*/*z* 191 and 217 mass chromatograms (Figure 4a,b) were compared with the Kuwaiti oil profiles reported by Arekhi,^52^ Wang et al.,^53^ Hauser et al.,^54^ Abdullah and Connan,^55^ Kruge et al.,^56^ and Peters et al.^46^ They all had a similar terpane distribution, with a maximum at H29, elevated H35/H34 ratio (H35 S and R over H34 S and R; varying from 0.9 to 1.0, Table S2), and a higher Tet24 abundance than the tricyclic terpanes, Tr19/Tr29 (Figure 4a). The one exception is the 18α-22,29,30-trisnorneohopane (Ts), which has a higher abundance than the 17α-22,29,30-trisnorhopane (Tm) in the 2023 samples, compared with the Kuwaiti oils reported in the studies cited above. The steranes also have a similar distribution among the samples, with the regular steranes being distributed as C29 > C27 > C28, while the diasteranes (e.g., Dia27S and Dia27R) are much less abundant (Figure 4b).

The fingerprints of the aromatic fractions of the four 2023 samples were all similar (Figure S8), with a marked UCM and high abundances for the C1 to C4 alkylated dibenzothiophenes (DBT1–4; see Figure S9), which is related to crude oils with a high sulfur content.^56^ Also in notable high abundance are the C29 and C30 monoaromatic 8,14-secohopanes, other petroleum biomarkers (SH29 and SH30, respectively; see their distribution in Figure S10 based on the identification by their mass spectra according to Wei and Songnian^57^). The C32–35 benzohopanes are also present (Figure S11; identification according to Wei and Songnian^57^).

The distribution of the aromatic compounds is similar to that of the Kuwaiti crude oil sample collected in 2014 (see Kruge et al.^56^) from an area affected by a vast oil spill that occurred during the first Gulf War. This oil was preserved from weathering for years by the dry and anoxic conditions of the desert region. Other significant similarities can be observed in the phenanthrene (P) and the alkylated phenanthrenes (P1 and P2) distribution (Figure S12), with a considerably greater abundance of 9-methylphenanthrene (9-MP), in comparison with its isomeric counterparts. Significant differences were only found at the beginning of the TICC (total ion current chromatograms), which encompass more volatile and soluble compounds, such as methylated naphthalenes (Figures S8 and S13). These compounds are more susceptible to weathering (see Kruge et al.^56^), so the differences likely reflect a longer environmental exposure time.

### Chemical Fingerprint of the 2023 Oil Spill

#### Genesis

**Figure 5:**
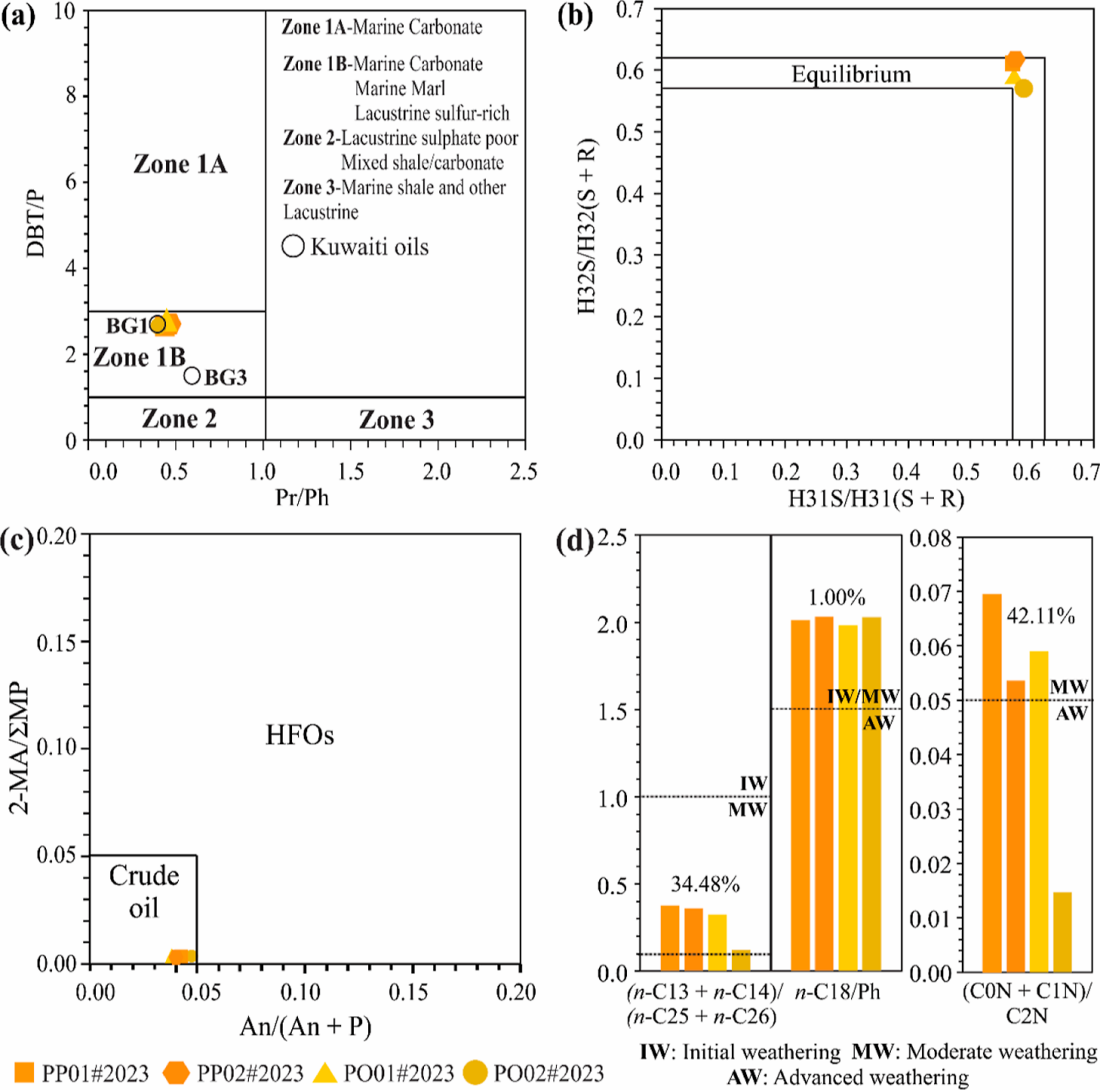
(a) Plot of Pr/Ph vs DBT/P for the depositional environmental assessment following Abdullah and Connan.^55^ (b) Plot of H31S/H31(S + R) vs H32S/H32(S + R) for the assessment of thermal maturity. (c) Plot of An/(An + P) vs 2-MA/ΣMP to assess the type of spilled oil. (d) Bar chart of the *n*-alkane, phytane, and naphthalene parameters to assess weathering stages.^59^ BG1 and BG3 = two oil samples from the Burgan field in Kuwait assessed by Abdullah and Connan^55^ HFOs = heavy fuel oils.

The genesis of the oil is a primary control of the oil fingerprint and should be understood in oil spill investigations.^21^ The geochemical parameters Pr/Ph and DBT/P, together with parameters Tr22/Tr21, Tr24/Tr23, Tr26/Tr25, H31R/H30, H29/H30, and H35S/H34S parameters, were used to assess the depositional environment of the source rock of the 2023 sample.^46,55^ The results indicate that the 2023 oil was generated from a source rock with carbonate lithology (Figures 5a and S14a–c; Table S5). In addition, the TPP/(TPP + Dia27) and H31R/H30 ratios (Figure S14d and Table S5) indicate a marine origin.^23,58^

The Pr/Ph and DBT/P ratios of the 2023 samples are also close to those of the two oil samples (BG1 and BG3) from the Burgan field in Kuwait (Figure 5a) reported by Abdullah and Connan.^55^ The Tr22/Tr21, Tr24/Tr23, Tr26/Tr25, H31R/H30, H29H/H30, and H35/H34 ratios (Figure S14a–c) of the 2023 samples are also similar to those of the oil from the Magwa field (Figure S14a–c) reported by Peters et al.^46^ These results confirm the close similarity between the 2023 samples and the crude oils from the Burgan field in Kuwait, which are sourced from a marine carbonate rock.^55^

The distribution of the regular steranes as C29 > C27 > C28 (Figure S3) is also consistent with carbonate source rocks,^56,60^ together with the high abundance of monoaromatic 8,14-secohopanes (Figure S10)^61^ and the complete C32–C35 benzohopane series (Figure S11).^56,62^

Thermal maturity was assessed by geochemical parameters using hopane and sterane isomers.^46^ The plot between the H31S/H31(S + R) and H32S/H32(S + R) ratios (Figure 5b) indicates that the principal generation phase was reached in the 2023 samples.^63^ However, the plot between the C29ααα 20S/(20S + 20R) and C29 ββ/C29 (ββ + αα) ratios points that the oil is immature (Figure S15a),^46^ which indicates that the oil was in the early stages of the oil window generation.

Maturity was also assessed based on the vitrinite reflectance (Rc) derived from the methylphenanthrene index (MPI-1; Radke;^64^ Rc = 0.60MPI-1 + 0.40). The values indicate immature to early mature oils (Table S6, Figure S15b), consistent with the low thermal maturity of the 2023 samples.

The plot between the C29ααα 20S/(20S + 20R) and C29 ββ/C29 (ββ + αα) ratios (Table S6) was also used to compare the maturity of the 2023 samples with that of the five Kuwaiti samples (Oil I, II, III, IV, and V) reported by Hauser et al.^54^ (Figure S15a). Only two of the Kuwaiti samples (III and V) were mature, based on the C29αα 20S/(20S + 20R) ratio, whereas all the C29 ββ/C29 (ββ + αα) ratios were typical of immature oils (Figure S15a).^46^

#### Refinery Processing

Oil spills in marine and coastal environments may be composed of crude oil or refined petroleum products.^65−68^ Crude oil may sometimes be confused with refined products, particularly heavy fuel oils, given that they share some physical and chemical properties.^69−71^

Anthracene and 2-methylanthracene, which are formed in abundance when crude oil is exposed to thermal alteration processes,^70,72,73^ were detected tentatively at very low abundance in all the samples (Figure S12), resulting in low values for the An/(An + P) ratio (0.04–0.05; Table S7) that indicate crude oil (<0.05; Figure 5c). The values for the 2-MA/ΣMP ratio were nearly zero (Table S7), which is also consistent with crude oil (<0.05; Figure 5c).^71^

In addition, the predominance of the 9-/4- and 1-MP methylphenanthrene isomers over the 3- and 2-MP isomers (Figure S12) confirms that the oil has not been refined. As the 3- and 2-MP isomers are more stable, thermally, they would be more abundant than the less stable 9-/4 and 1-MP isomers if the oil had undergone some process of thermal alteration.^11,70^ In this case, the values obtained for the (3- + 2-MP)/(9-/4- + 1-MP) ratio (0.63–0.65; Table S7) are consistent with crude oil, i.e., <1.50 (Figure S16).^71^

#### Weathering

Weathering processes (which can be physical, chemical, or biological) tend to modify the composition of oil exposed to the environment over time and must be considered systematically in any oil spill investigation.^74−76^ The detection of the less resistant *n*-C13 to *n*-C22 alkanes in high abundance in the 2023 samples (Figures 3a and S1) indicates limited biological degradation, which is consistent with a recent release (days to weeks) of the oil.^74^ The *n*-alkanes lower than *n*-C13 (Figure S1) and naphthalene were not detected, probably because they evaporated soon after the oil spill,^74,77^ although this may also reflect the typical loss of volatile compounds during the extraction, concentration, and fractioning of the oil.

The Pr/Ph, Pr/*n*-C17, and Ph/*n*-C18 ratios are comparable to those of the Kuwaiti crude oil described by Peters et al.^46^ (Table S8), with an RSD lower than 14% considering all five samples, which is consistent with low biological degradation. However, the *n*-C27/*n*-C17 ratio varied considerably between the 2023 samples (0.34–0.42) and the Kuwaiti oil (0.24; Table S8), which indicates that evaporation had a considerable effect on the short-chain *n*-alkanes. The MPI-1 (0.67–0.68) and MDBTI-1 (1.43–1.47) ratios had an RSD of less than 7%, in comparison with the two samples (BG1 and BG3) from the Greater Burgan Oil Field (Table S9),^55^ as well as a DBT/P ratio (2.21–2.79) with an RSD of 17.27% (Table S9), which indicate that weathering was not extensive enough to have affected the phenanthrenes, dibenzothiophenes, or their alkylated compounds significantly.

We also applied the classification proposed by Yim et al.,^59^ who assessed Kuwaiti, Iranian, and Emirati oils from the Hebei Spirit oil spill on the coast of the Republic of Korea, to tentatively assess the weathering stage of the 2023 samples. This classification has four categories: I (initial weathering—IW); II (moderate weathering—MW); III (advanced weathering—AW); and IV (extreme weathering—EW). The *n*-C18/Ph ratio ranges from 1.98 to 2.03 (RSD = 1.0%), which is characteristic of initial to moderate weathering (Figure 5d, Table S10).^59,74^ The (*n*-C13 + *n*-C14)/(*n*-C25 + *n*-C26) and (C0N + C1N)/C2N ratios (0.12–0.38, RSD = 34.48%; 0.01–0.07, RSD = 42.11%, respectively, except for sample PO02#2023; Figure 5d, Table S10) are also consistent with moderate weathering, given that they reflect the processes of dissolution and evaporation. The high RSD values observed here are due to the influence of the PO02#2023 sample, which had undergone an atypical loss of volatile compounds compared to the other 2023 samples.

The moderate weathering (II) classification of the 2023 samples indicates that the oil was released at least 10 days and up to three months before to its arrival on the beaches of Salvador.^59^ This upper limit can be reduced to 19 days, however, considering the similarity of the PAH profile of the 2023 samples (naphthalene, fluorene, phenanthrene, dibenzothiophene, chrysene, and their alkyl homologues; Figure S17), which were exposed to warm tropical conditions, with sample S1–2 of the Hebei Spirit oil spill, collected after 19 days by Yim et al.^59^ in temperate winter conditions. The detection of methylnaphthalenes (N1) and fluorene (F) (Figure S17) is also consistent with a short-term oil spill.

### Ocean Circulation

The south equatorial current flows southeast to northwest across the Tropical South Atlantic Ocean, where its southern branch (sSEC) approaches the continental margin of Brazil and bifurcates, forming the northward-flowing North Brazil under-current and the southward-flowing Brazil current (BC). The location of this bifurcation oscillates from approximately 19°S in May–June to around 10°S in November–December. Mesoscale eddies, which are circular currents, are typically present within the area of this bifurcation. The sSEC current system could have transported the 2023 oil spill toward the coast of Bahia, given that it usually transports materials from the entire eastern Tropical Atlantic toward the Brazilian continental shelf, from where it will either continue northwestward on the NBC or southwestward, on the BC.^35^

However, as the weathering analyses indicate that the oil entered the ocean only a few days to a few weeks before it reached the coast, its source was relatively close to the coast. On September 10, 2023, two eddies were present in the region, one at around 16.0°S, 36.5°W, and the other near 13.5°S, 34.5°W (Figure S18a). In the region to the north of Salvador, these two eddies would have advected water toward the coast at 0.25 m s^–1^ (21.6 km day^–1^) between September 10 and 14th, 2023 (Figure S18b,c). As no oil was found to the north of Salvador, the potential source would have to have been located to the east or southeast of Salvador, probably within the Brazilian Exclusive Economic Zone, which is subject to intense oil tanker traffic in the South Atlantic Ocean. Any oil dumped in this area has a high probability of reaching the shore.^18^ On September 15, 2023, the eddies weakened, and the circulation along the continental shelf veered southwestward (Figure S18d), which would account for the subsequent arrival of the tarballs on the beaches to the south of Salvador.

### Implications

Forensic environmental geochemistry combined with the ocean current circulation patterns assessment revealed that the latest oil spill affecting the Brazilian coast, in September 2023, likely originated from a tanker transporting Middle Eastern crude oil through the South Atlantic Ocean, off Brazil. These findings further highlight the vulnerability of the country’s northeastern coast to the dumping of oil in offshore shipping routes in the South Atlantic Ocean and the lack of adequate monitoring, even in inshore areas.

## Supporting Information Available

Additional details on the contextualization of the oil spill and the extraction and fractionation of the oil, diagnostic ratios applied in the analyses and the parameters based on the saturated and aromatic compounds, selected ion chromatograms, GC-FID chromatograms, the map of the maritime crude oil transportation routes in the South Atlantic Ocean, and additional graphs that provide more details of the results (PDF)
