# Supplementary material for: Forensic Geochemistry Reveals International Ship Dumping as a Source of New Oil Spill in Brazil’s Coastline (Bahia) in Late 2023
Source: Environ Sci Technol. 2024 May 13;58(21):9328–38. doi: 10.1021/acs.est.4c01520 (PMC11152363; doi:10.1021/acs.est.4c01520)
Supplement: Supplementary file 1 — es4c01520_si_001.pdf [file es4c01520_si_001.pdf]

Supporting Information

Forensic Geochemistry Reveals International Ship Dumping as a Source of New Oil Spill in Brazil's Coastline (Bahia) in Late 2023

Laercio L. Martins[*,†,§], *Vinícius B. Pereira*[‖], *Adriana P. Nascimento*[§], *Rufino Neto A. Azevedo*[‡], *André H. B. Oliveira*[§,‡], *Carlos Eduardo P. Teixeira*[§], *Débora A. Azevedo*[‖], *Georgiana F. da Cruz*[†], *Rivelino M. Cavalcante*[§], *Tommaso Giarrizzo*[§]

[†]Laboratory of Petroleum Engineering and Exploration (LENEP), North Fluminense State University (UENF), Macaé, Rio de Janeiro, Brazil

[§]Institute of Marine Sciences (LABOMAR), Federal University of Ceará (UFC), Fortaleza, Ceará, Brazil

[‖]Institute of Chemistry (IQ), Federal University of Rio de Janeiro (UFRJ), Rio de Janeiro, Brazil

[‡]Chemistry and Physical Chemistry Department (DQAFQ), Federal University of Ceará, Fortaleza (UFC), Ceará, Brazil

KEYWORDS: Oil Dumping; Tarballs; International Waste; Marine Pollution; Northeastern Brazil; Environmental Geochemistry.

Corresponding author:

E-mail address: laercio@lenep.uenf.br (L.L. Martins)

The Supporting Information includes 28 pages, 10 tables, and 18 figures.

Table of Contents

CONTEXTUALIZATION OF THE OIL SPILL 3
SUPPORTING METHOD DETAILS 4
Table S1........ 5
Table S2........ 5
Table S3........ 6
Table S4........ 8
Table S5........ 9
Table S6........ 9
Table S7........ 9
Table S8........ 9
Table S9........ 10
Table S10........ 10
Figure S1. 11
Figure S2 12
Figure S3 13
Figure S4 14
Figure S5 15
Figure S6 16
Figure S7 17
Figure S8 18
Figure S9 19
Figure S10 20
Figure S11 21
Figure S12 22
Figure S13 23
Figure S14 24
Figure S15. 24
Figure S16 25
Figure S17 25
Figure S18 26
References 27

CONTEXTUALIZATION OF THE OIL SPILL

Tarballs were first found on September 10th, 2023, on at least nine beaches in the city of Salvador, capital of the Brazilian state of Bahia, including Amaralina, Pituaçu, Ondina, Paciência, Corsário, Jardim de Alah, Jaguaribe, Patamares, Piatã, Rio Vermelho, and Barra beaches.[1,2] Three days after their first appearance, the tarballs had migrated south, where they were recovered from Guaibim beach, in the municipality of Valença, approximately 75 km south of Salvador.[3,4] On September 14th and 15th, tarballs were also found further south, in the municipality of Cairú.[5] Oil was also found at Cacha Prego, in the municipality of Vera Cruz, Garcez beach and Rio da Dona, in Jaguaripe, and Morro de São Paulo and Boipeba island, in Cairú.[2] The 2023 oil spill extended at least 120 km of coastline, from Piatã beach to Boipeba island (**Figure 1**).

The Bahia state government coordinated the collection of the tarballs, together with the Fire Department, Civil Defense, the State Secretariat for the Environment (SEMA), and the State Institute for the Environment and Water Resources (INEMA), combining efforts to clean the beaches and dispose of the contaminants adequately.[2] According to these authorities, the full extent of the damage was difficult to measure, but the contamination occurred primarily on the beaches and in the coastal zone, except for an oil stain near Morro de São Paulo.[2]

A sample collected from Barra beach, Salvador, was analyzed by researchers from the Federal University of Bahia, who stated that the composition of the oil did not correspond to that of the oils that beached on the coast of northeastern Brazil in 2019 and late 2022.[4] The characteristics of the sample aligned closely with those of oil produced in the basins of Kuwait. The authors suggested that the tarballs likely originated from a discharge of contaminated water from an oil tanker moving across the sea off Bahia.[4] However, their assessment was restricted to a single sample from Barra beach, and they did not evaluate the factors that may have affected the tarballs before they reached the beach.

SUPPORTING METHOD DETAILS

Oil extraction and separation. The oil extraction of the 2019, the 2022.1 and 2022.2 events was performed from 1.0 g of oiled materials (oil mixed with sediment) using 5 mL of dichloromethane as a solvent, which was stirred, centrifuged, and the organic phase was relocated to a flask. This procedure was replicated four more times to maximize oil extraction. The solvent with the oil was roto-evaporated.[6] The asphaltene precipitation was performed from 300 mg of extracted oil by 100 μL of dichloromethane mixed with 12 mL of *n*-hexane through an ultrasonic bath for 10 min, followed by centrifugation.[7] This was intended to break the stability of the resin and asphaltene colloids in the oil and then separate the asphaltene and the maltene fractions. The maltene fraction dissolved in the solvent was removed, and the precipitated asphaltene was washed five times with 12 mL of *n*-hexane to ensure maximum recovery of the maltene,[8] then roto-evaporated. Finally, approximately 40 mg of maltene were fractionated in saturated, aromatics, and resins by liquid column chromatography, where each fraction was eluted with 30 mL of *n*-hexane, *n*-hexane:dichloromethane (8:2, *v:v*), and dichloromethane:methanol (9:1, *v:v*), respectively. After collecting each fraction, they are taken to the rotary evaporator to remove the solvents.[7] All solvents were chromatographic/HPLC grade from Sigma-Aldrich Chemical.

The 2023 oils were extracted using an ultrasonic bath of approximately 2.0 g of sediment, using a mixture of dichloromethane:methanol (9:1) as extraction solvent (3 × 50 mL). Bench procedures were carefully conducted as previously described. After solvent removal, asphaltene precipitation was performed from 100 mg of extracted oil, diluted in 5 mL of *n*-hexane, stirred for 5 minutes, and left standing for 24 h.[9] After that, the flasks were centrifuged for 5 minutes, and asphaltenes were separated by decantation. The flasks were washed four more times with 2 mL hexane to completely separate the maltenes, which were then fractionated into saturated, aromatic, and resins.[10]

Table S1. Sample codes, collection date, and locality information, including the beach, state, and coordinates, of the spilled oil samples

Sample code	Collection date	Locality		
		Beach	State	UTM Coordinates
PP01#2023	11/09/2023	Paciência	Bahia	-13.011023, -38.496700
PP02#2023	11/09/2023	Paciência	Bahia	-13.011023, -38.496700
PO01#2023	11/09/2023	Ondina	Bahia	-13.010796, -38.503498
PO02#2023	11/09/2023	Ondina	Bahia	-13.010796, -38.503498
P01#2022.2	01/10/2022	Futuro	Ceará	-3.763056, -38.439444
P02#2022.2	24/09/2022	Icaraí	Ceará	-3.67444444, -38.6644444
P04#2022.1	28/01/2022	Cumbe	Ceará	-4.472713, -37.740430
P05#2022.1	28/01/2022	Canoa Quebrada	Ceará	-4.519647, -37.7007260
P01#2019	01/11/2019	Mangue do Rio Jaguaribe	Ceará	-4.500947, -37.724562
P03#2019	07/11/2019	Paracuru	Ceará	-3.399421, -39.011706

Table S2. Part 1: Diagnostic ratios based on tricyclic and pentacyclic terpanes (*m/z* 191), steranes (*m/z* 217), triaromatic steroids (*m/z* 231), and tetracyclic polyprenoids (*m/z* 259) for similarity assessment[11]

Diagnostic Ratios	PP01#2023	PP02#2023	PO01#2023	PO02#2023	P01#2022.2	P02#2022.2	P04#2022.1	P05#2022.1	P01#2019	P03#2019
[1]H29/H30	1.26	1.27	1.14	1.28	0.93	0.93	0.76	0.77	0.66	0.68
[2]Gam/H30	0.04	0.04	0.04	0.04	0.06	0.05	0.09	0.10	0.13	0.12
[3]Tr20/Tr23	0.25	0.24	0.24	0.23	0.29	0.29	0.10	0.07	0.15	0.16
[4]Tr21/Tr23	0.28	0.28	0.29	0.29	0.40	0.42	0.27	0.22	0.32	0.29
[5](Tr28 + Tr29)/H30	0.10	0.10	0.09	0.11	0.19	0.19	0.17	0.17	0.63	0.64
[6]Steranes/Hopanes	0.13	0.13	0.14	0.13	0.29	0.30	0.13	0.12	0.25	0.23
[7]H31R/H30	0.46	0.47	0.46	0.48	0.45	0.44	0.40	0.41	0.45	0.44
[8]TA C26 S/(TA C26R + TA C27S)	0.11	0.11	0.11	0.12	0.12	0.13	0.19	0.20	0.17	0.18
[9]Tr26/Tr23	0.26	0.25	0.28	0.27	0.39	0.40	0.52	0.54	0.30	0.30
[10]H35/H34	0.97	0.96	0.96	0.86	1.01	0.94	1.01	1.01	0.98	1.01
[11]TA C28R/C28S	0.82	0.85	0.86	0.88	0.97	0.93	1.01	0.97	1.04	1.06
[12]TA C27R/C28R	0.71	0.71	0.71	0.70	1.17	1.16	0.91	0.90	1.14	1.12
[13]Tr26/Tr25	0.70	0.67	0.64	0.75	0.69	0.66	0.99	1.06	0.66	0.68
[14]TPP/(TPP + Dia27)	0.43	0.43	0.41	0.42	0.39	0.39	0.59	0.59	0.81	0.82
[15]Ts/Tm	1.00	1.02	1.05	1.03	0.53	0.51	0.45	0.45	0.49	0.47
[16]TA 28S/(26R + 27S)	1.14	1.17	1.13	1.15	0.63	0.65	0.78	0.81	0.54	0.54

[1]C29 hopane/C30 hopane; [2]Gammacerane/C30 hopane; [3]C20 tricyclic terpane/C23 tricyclic terpane; [4]C21 tricyclic terpane/C23 tricyclic terpane; [5][C28 tricyclic terpane (S + R) + C29 tricyclic terpane (S + R)]/C23 tricyclic terpane; [6]ΣC27-C9 steranes/ΣC29-C35 hopanes; [7]C31 hopane R/C30 hopane; [8]C26 S triaromatic steroid/(C26 R triaromatic steroid + C27 S triaromatic steroid); [9]C26 tricyclic terpane (S + R)/C23 tricyclic terpane; [10]C35 hopane (S + R)/C34 hopane (S + R); [11]C28 R triaromatic steroid/C28 S triaromatic steroid; [12]C27 R triaromatic steroid/C28 R triaromatic steroid; [13]C26 tricyclic terpane/C25 tricyclic terpane; [14](C30 tetracyclic

polyprenoids/[C30 tetracyclic polyprenoids + C27diasterane βα (S + R)]; [15]C27 18α-trisnorneohopane/C27 17α-trisnorhopane; [16]C28 S triaromatic steroid/(C26 R triaromatic steroid + C27 S triaromatic steroid).

Table S2. Part 2: The relative standard deviation (RSD) of the four 2023 oil samples, the four 2023 and two 2022.2 oil samples, the four 2023 and two 2022.1 oil samples, and the four 2023 and two 2019 samples, respectively

Diagnostic Ratios	[17]RSD 2023 (%)	[18]RSD 2022.2 (%)	[19]RSD 2022.1 (%)	[20]RSD 2019 (%)
[1]H29/H30	4.6	13.3	21.0	25.9
[2]Gam/H30	3.5	15.6	41.6	55.4
[3]Tr20/Tr23	2.1	8.9	40.4	19.4
[4]Tr21/Tr23	2.5	18.6	8.8	5.5
[5](Tr28 + Tr29)/H30	4.2	32.2	27.1	90.2
[6]Steranes/Hopanes	4.4	40.1	4.7	29.1
[7]H31R/H30	1.8	2.8	7.2	3.0
[8]TA C26 S/(TA C26R + TA C27S)	1.8	5.1	27.6	22.3
[9]Tr26/Tr23	3.6	20.0	35.7	6.6
[10]H35/H34	4.8	4.9	5.3	4.8
[11]TA C28R/C28S	2.3	5.5	7.4	10.4
[12]TA C27R/C28R	0.7	2.4	12.2	23.7
[13]Tr26/Tr25	6.0	5.1	20.1	5.2
[14]TPP/(TPP + Dia27)	2.6	4.2	16.7	33.3
[15]Ts/Tm	2.0	27.7	32.7	30.5
[16]TA 28S/(26R + 27S)	1.2	24.4	16.2	30.3

[17]RSD of the 2023 oil samples; [18]RSD of the 2022.2 and the 2023 oil samples; [19]RSD of the 2022.1 and the 2023 oil samples; [20]RSD of the 2019 and the 2023 oil samples.

Table S3. Values of diagnostic ratios based on the recalcitrant tricyclic and pentacyclic terpanes and steranes of the four 2023 spilled oils used in the comparison with the same ratios of crude oils from the main countries exporters of crude oil from the Middle East, Africa, and Latin American presented in Peters et al.[12] The average of the diagnostic ratios used in **Figure 4c** and **d** are also presented, in addition to their relative standard deviation (RSD)

Samples	PP01#2023	PP02#2023	PO01#2023	PO02#2023	Average	RSD (%)
[1]Tr19/Tr23	0.17	0.17	0.20	0.15	0.17	12.0
[2]Tr22/Tr21	0.99	0.94	0.95	1.03	0.98	3.5
[3]Tr24/Tr23	0.37	0.35	0.39	0.35	0.37	4.8
[4]Tr26/Tr25	0.70	0.67	0.64	0.75	0.69	6.0
[5]Tet24/Tr23	1.30	1.22	1.21	1.26	1.25	2.8
[6]H29/H30	1.26	1.27	1.14	1.28	1.24	4.6
[7]H31R/H30	0.46	0.47	0.46	0.48	0.47	1.8
[8]Gam/H31R	0.09	0.09	0.09	0.09	0.09	1.8
[9]H35S/H34S	0.98	0.98	0.93	0.83	0.93	6.5
[10]Steranes/Terpanes	0.13	0.13	0.14	0.13	0.13	2.7
[11]H29 20S/R	0.88	0.87	0.73	0.75	0.81	8.2
[12]Ts/Tm	1.00	1.02	1.05	1.03	1.02	2.0
[13]%C27	35.67	35.16	35.78	35.08	35.42	0.9

**[14]%C28**	16.98	16.41	18.00	17.27	17.17	3.3
**[15]%C29**	47.36	48.42	46.22	47.65	47.41	1.7

[1]C19 tricyclic terpane/C23 tricyclic terpane; [2]C22 tricyclic terpane/C21 tricyclic terpane; [3]C24 tricyclic terpane/C23 tricyclic terpane; [4]C26 tricyclic terpane (S + R)/C25 tricyclic terpane; [5]C24 tetracyclic terpane/C23 tricyclic terpane; [6]C29 hopane/C30 hopane; [7]C31 hopane R/C30 hopane; [8]Gammacerane/C31 hopane R; [9]C35 hopane S/C34 hopane S; [10]ΣC27-C9 steranes/ΣC29-C35 hopanes; [11]C29 hopane S/C29 hopane R; [12]C27 18α-trisnorneohopane/C27 17α-trisnorhopane; [13]ΣC27 steranes/ΣC27-C29 steranes; [14]ΣC28 steranes/ΣC27-C29 steranes; [15]ΣC29 steranes/ΣC27-C29 steranes.

Table S4. Geological information of the petroleum from the main countries exporters of crude oil from the Middle East, Africa, and Latin American (information from Peters et al.[12])

Oil	Country	Basin	Reservoir rock			Source rock		Reference (Peters et al.[12])
			Field	Formation	Period	Formation/Member	Period	
**SA(1)**	Saudi Arabia	Western Platform	Raghib	Unayzah	Permian	Qusaiba (Western Platform)	L. Silurian	Page 793
**SA(2)**	Saudi Arabia	Central Arabian	Bakr	Hanifa	U. Jurrasic	Tuwaiq Mountain/Hanifa	Jurassic (Callovian-Oxfordian)	Page 865
**Ir(1)**	Iran	Dezful Embayment	Karun	Asmari	Oligocene	Pabdeh	Tertiary (Eocene)	Page 929
**Ir(2)**	Iran	Dezful Embayment	Marun	Asmari	Oligocene	Kazhdumi	M. Cretaceous	Page 868
**Ku**	Kuwait	Burgan Rumaila High	Magwa	Burgan	M. Cretaceus	Najmah	Jurassic (Oxfordian)	Page 863
**UAE(1)**	United Arab Emirates	Southern Arabian Gulf	Zakum	Shilaif	M. Cretaceus	Shilaif	Cretaceous	Page 869
**UAE(2)**	United Arab Emirates	Southern Arabian Gulf	Upper Zakum	Thamama	L. Cretaceus	Diyab	Jurassic	Page 866
**Om(1)**	Oman	South Oman	Marmul	Haushi	Permian	Huqf	Cambrian	Page 773
**Om(2)**	Oman	South Oman	Sayyala	Gharif	Permian	Dhahaban	Cambrian	Page 775
**An**	Angola	Kwanza	Quenguela	Quifangondo	Miocene	Iabe	U. Cretaceous	Page 854
**Ni**	Nigeria	Niger Delta	Meren	-	Miocene	Akata/Agbada	Tertiary	Page 951
**Li**	Libya	Sirte	Ora	B	Cretaceous	Sirte	U. Cretaceous	Page 914
**Al**	Algeria	Trias	Hassi Messaoud	R-1	Cambrian	Tanezzuft (Shale)	L. Silurian	Page 792
**Br(1)**	Brazil	Recôncavo	Candeias	Candeias	L. Cretaceous	Gomo	L. Cretaceous	Page 842
**Br(2)**	Brazil	Campos	Garoupa	Macaé	Albian	Lagoa Feia	Cretaceous (Hauterivian-Aptian)	Page 847
**Br(3)**	Brazil	Ceará	Curimã	Açu	Aptian	Alagamar	Cretaceous (Aptian)	Page 850
**Co**	Colombia	Upper Magdalena	Gigante	Guadalupe	Cretaceous	Villeta	U. Cretaceous	Page 913
**Ve**	Venezuela	Maracaibo	Bachaquero	-	-	La Luna	U. Cretaceous	Page 911
**Ga(1)**	Gabon	Congo	Rabi Kounga	Gamba	Aptian	Kissenda/Melania lacustrine shales	Cretaceous (Neocomian)	Page 848
**Ga(2)**	Gabon	Gabon	Port Gentil Ocean	Anguille	Senonian	Azile	Cretaceous (Turonian)	Page 852

Peters, K.E., Walters, C.C., Moldowan, J.M., 2005. The Biomarker Guide: Biomarkers and Isotopes in the Petroleum Exploration and Earth History, 2nd ed., University Press: Cambridge, vol. 2.

Table S5. Ratios using the isoprenoids (pristane and phytane), dibenzothiophene (*m/z* 184), and terpanes (*m/z* 191) to assess depositional environmental

Ratio	PP01#2023	PP02#2023	PO01#2023	PO02#2023	[a]BG1	[a]BG3	[b]Magwa
[1]Pr/Ph	0.44	0.47	0.45	0.41	0.43	0.59	-
[2]DBT/P	2.73	2.71	2.79	2.72	2.72	1.56	-
[3]Tr22/Tr21	0.99	0.94	0.95	1.03	-	-	1.02
[4]T24/T23	0.37	0.35	0.39	0.35	-	-	0.32
[5]Tr26/Tr25	0.70	0.67	0.64	0.75	-	-	0.68
[6]H31R/H30	0.46	0.47	0.46	0.48	-	-	0.46
[7]H29H/H30	1.26	1.27	1.14	1.28	-	-	1.20
[8]H35S/H34S	0.98	0.98	0.93	0.83	-	-	1.07
[9]TPP/(TPP + Dia27)	0.43	0.43	0.41	0.42	-	-	-

[1]Pristane/Phytane; [2]Dibenzothiophene/Phenanthrene; [3]C22 Tricyclic terpane/C21 Tricyclic terpane; [4]C24 Tricyclic terpane/C23 Tricyclic terpane; [5]C26 Tricyclic terpane (S + R)/C25 Tricyclic terpane; [6]C31 17α(H),21β(H)-Homohopane (22R)/C30 17α(H),21β(H)-Hopane; [7]C29 17α(H),21β(H)-30-Norhopane/C30 17α(H),21β(H)-Hopane; [8]C35 17α(H),21β(H)-30,31,32,33,34-Pentakishomohopane(22S)/C34 17α(H),21β(H)-30,31,32,33-Tetrakishomohopane (22S); [9]C30 Tetracyclic Polyprenoid 18α(H)/[C30 Tetracyclic Polyprenoid 18α(H) + C27 (S + R) diasteranes].

[a]Crude oil samples from the Burgan field in Kuwait assessed by Abdullah and Connan.[13]

[b]Crude oil sample presented in Peters et al.[12]

Table S6. Terpanes (*m*/z 191) and steranes (*m/z* 217) ratios to assess thermal maturity

Ratio	PP01#23	PP02#2023	PO01#2023	PO02#2023	*Oil I	*Oil II	*Oil III	*Oil IV	*Oil V
[1]H31S/H31(S + R)	0.57	0.58	0.58	0.58	-	-	-	-	-
[2]H32S/H32(S + R)	0.61	0.61	0.58	0.57	-	-	-	-	-
[3]Rc	0.80	0.81	0.79	0.82	-	-	-	-	-
[4]C29αα 20(s)/C29αα (20 S + R)	0.47	0.42	0.43	0.46	0.51	0.51	0.53	0.50	0.54
[5]C29 ββ/C29 (ββ+αα)	0.44	0.47	0.48	0.50	0.56	0.56	0.58	0.57	0.59

[1]17α(H),21β(H)-30-Homohopane(S)/17α(H),21β(H)-30-Homohopane(S+R); [2]17α(H),21β(H)-30, 31-Bishomohopane(S)/17α(H),21β(H)-30, 31-bishomohopane(S + R); [3]Rc = 0.40 + 0.60 MPI 1; [4]29ααα 20(S) sterane/C29ααα (20 S + R) steranes; [5]C29ββ/(C29ββ + C29 αα) (S + R) steranes.

*Crude oils of Kuwait assessed by Hauser et al.[14]

Table S7. Ratios using the anthracene and phenanthrene (*m/z* 178), and the isomers of methylphenanthrene and the 2-methylanthracene (*m/z* 192) to assess the type of oil

Ratio	PP01#2023	PP02#2023	PO01#2023	PO02#2023
1An/(An + P)	0.04	0.04	0.04	0.05
[2]2-MA/ΣMP	0.00	0.00	0.00	0.00
[3](3- + 2-MP)/(9/4- + 1-MP)	0.65	0.65	0.63	0.63

[1]Anthracene/(Anthracene + Phenanthrene); [2]2-Methylanthrancene/Σmethylphenanthrene isomers; [3](3- + 2-methylphenanthrene)/(9/4- + 1- methylphenanthrene).

Table S8. Ratios using the *n*-alkanes and isoprenoids (pristane and phytane) to assess weathering processes

Ratio	*Kuwait oil	PP01#2023	PP02#2023	PO01#2023	PO02#2023	RSD
[1]Pr/Ph	0.66	0.44	0.47	0.45	0.41	13.38

[2]Pr/*n*-C17	0.25	0.21	0.22	0.23	0.21	6.14
[3]Ph/*n*-C18	0.40	0.50	0.49	0.50	0.49	9.70
[4]*n*-C27/*n*-C17	0.24	0.34	0.39	0.42	0.40	27.09

[1]Pristane/Phytane; [2]Pristane/C17 *n*-alkane; [3]Phytane/C18 *n*-alkane; [4]C27 *n*-alkane/C17 *n*-alkane
*Magwa Field[12]

Table S9. Ratios using the polycyclic aromatics compounds to assess weathering processes

Ratio	[a]BG1	[a]BG3	PO01#2023	PO02#2023	PP01#2023	PP02#2023	RSD
[1]MPI-1	0.59	0.66	0.67	0.68	0.67	0.68	4.75
[2]MDBTI-1	1.40	1.70	1.44	1.47	1.43	1.44	6.84
[3]DBT/P	2.72	1.56	2.79	2.72	2.73	2.71	17.27

[1]1.5 (2- + 3- methylphenanthrene)/(phenanthrene + 1- + 9- methylphenanthrene); [2]1.5 (4- + 2,3-Methyldibenzothiophene)/(Dibenzothiophene + 2,3- + 1- Methyldibenzothiophene); [3]Dibenzothiophene/Phenanthrene.

[a]Crude oil samples from the Burgan field in Kuwait assessed by Abdullah and Connan.[13]

Table S10. Ratios using the *n*-alkanes, phytane, naphthalene and their methylated series, to assess the stage of weathering

Weathering	Ratio	PP01#2023	PP02#2023	PO01#2023	PO02#2023	RSD
Evaporation	[1](*n*-C13 + *n*-C14)/(*n*-C25 + *n*-C26)	0.38	0.36	0.32	0.12	34.48
Evaporation + Disssolution	[2](C0N + C1N)/C2N	0.06	0.01	0.07	0.05	42.11
Biodegradation	[3]*n*-C18/Ph	2.01	2.03	1.98	2.03	1.00

[1](C13 *n*-alkane + C14 *n*-alkane)/(C25 *n*-alkane + C26 *n*-alkane); [2](Naphthalene + Methylnaphthalene)/Dimethylnaphthalene; [3]C18 *n*-alkane/Phytane.

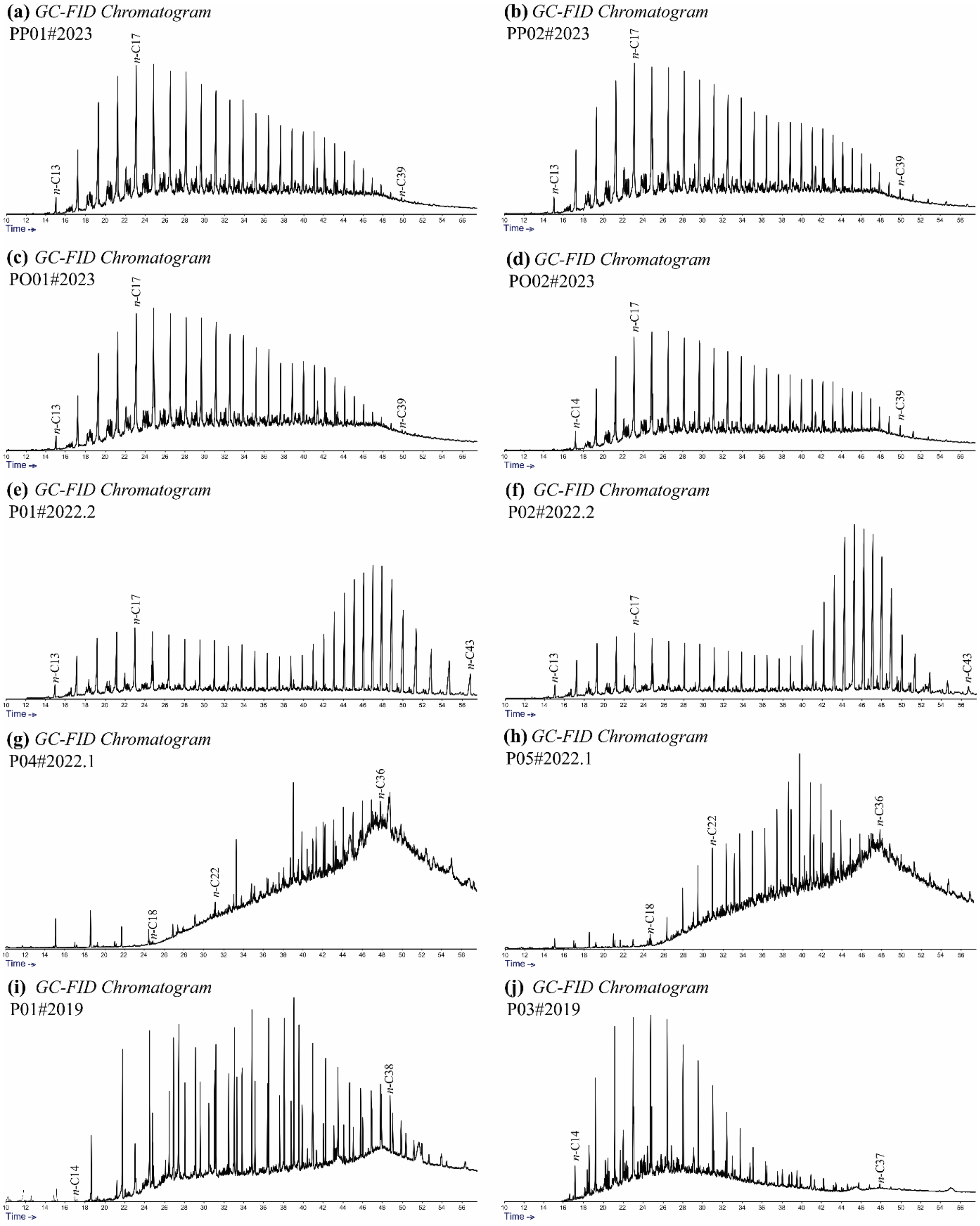

Figure S1. (a-d) GC-FID chromatograms of the saturated fractions of the four 2023 spilled oils, in addition to (e, f) the two tarballs collected in late 2022, (g, h) the two spilled oils collected in early 2022, and (i, j) the two spilled oils collected in 2019.

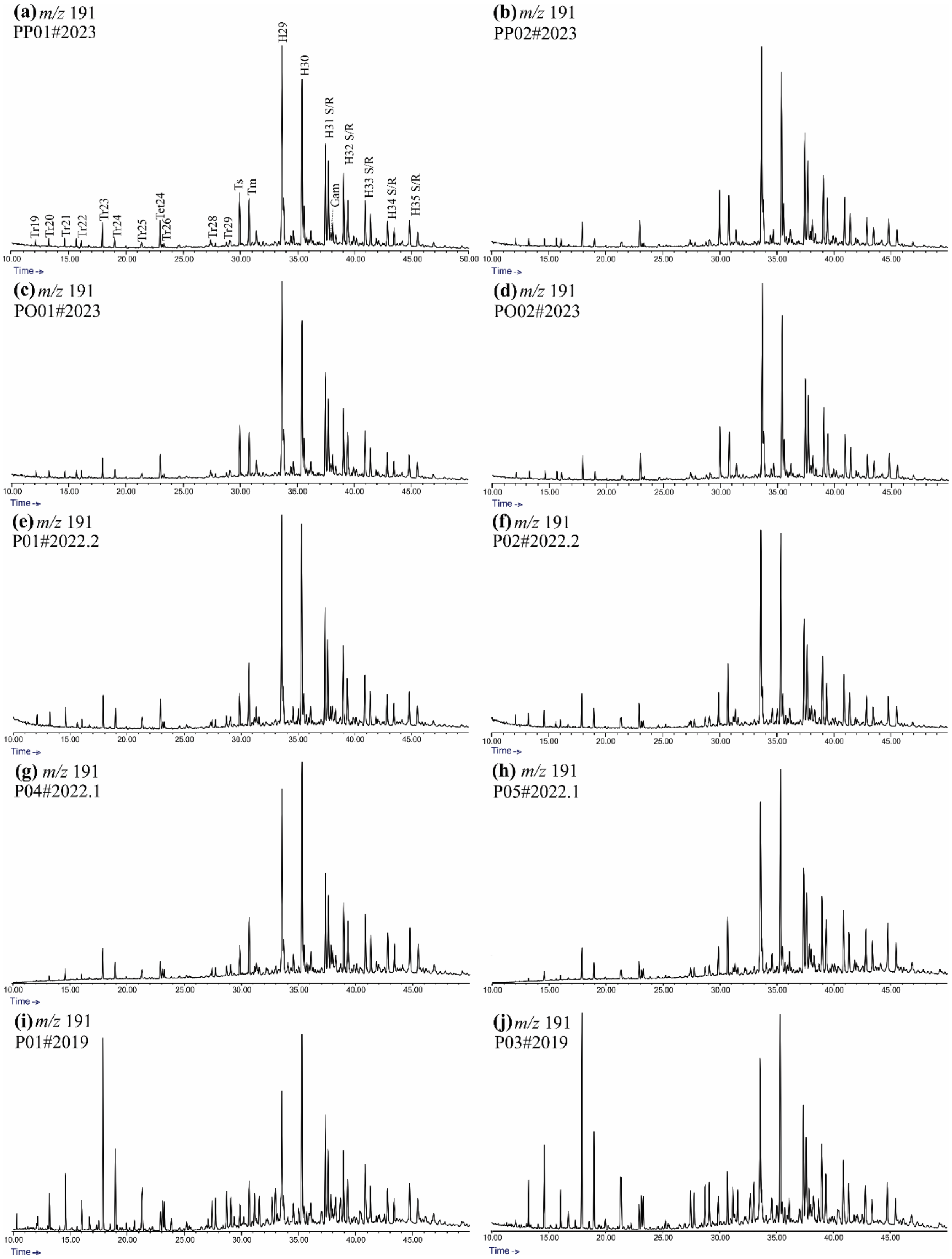

Figure S2. Selected ion chromatograms *m/z* 191, showing the terpanes distribution, of the four 2023 spilled oil at the State of Bahia (a-d), in addition to the two tarballs collected in late 2022 (e, f), the two spilled oils collected in early 2022 (g, h), and the two spilled oils collected in 2019 (i, j).

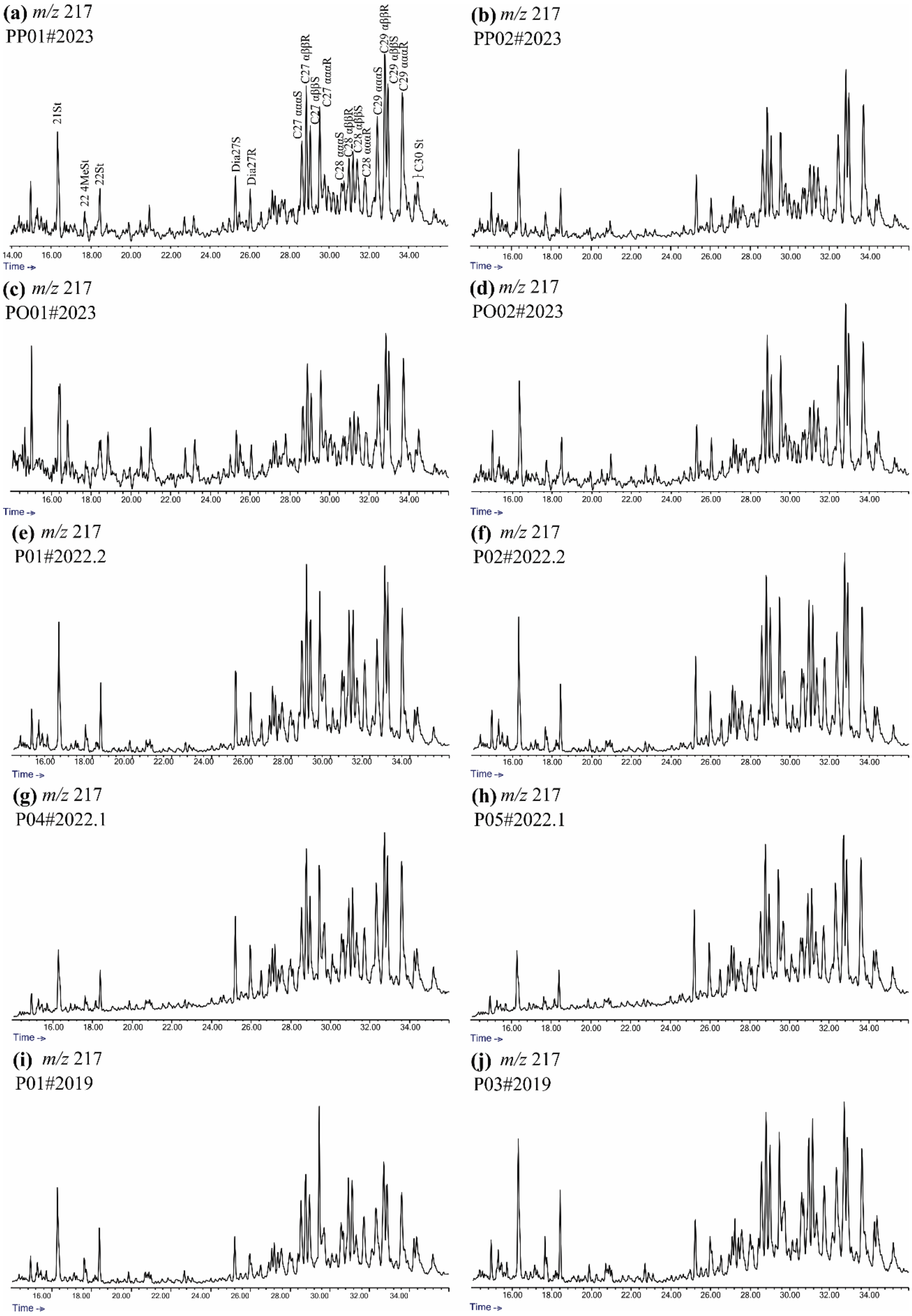

Figure S3. Selected ion chromatograms *m/z* 217, showing the steranes distribution, of the four 2023 spilled oil at the State of Bahia (a-d), in addition to the two tarballs collected in late 2022 (e, f), the two spilled oils collected in early 2022 (g, h), and the two spilled oils collected in 2019 (i, j).

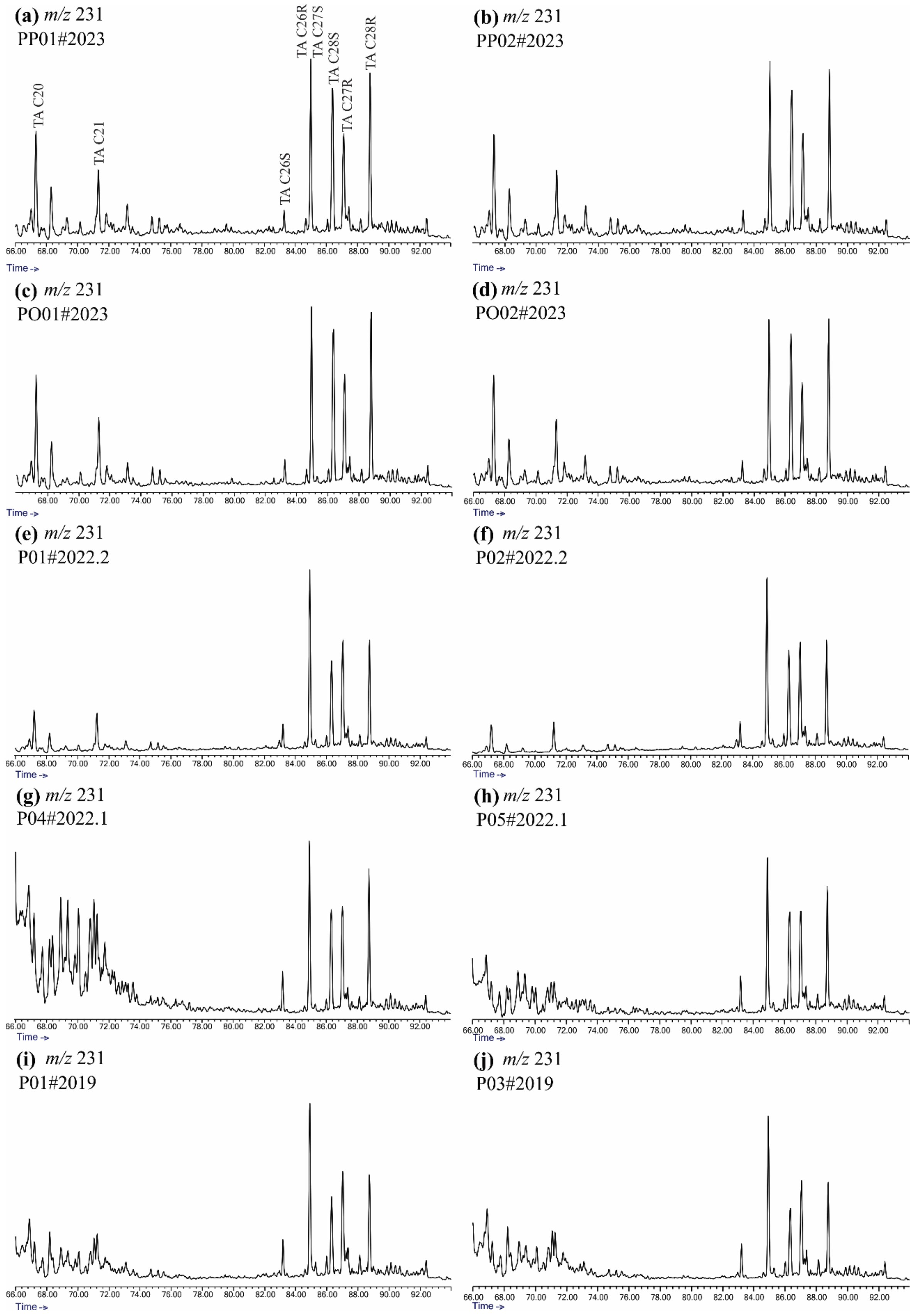

Figure S4. Selected ion chromatograms *m/z* 231, showing the triaromatic steranes (TAS) distribution, of the four 2023 spilled oil at the State of Bahia (a-d), in addition to the two tarballs collected in late 2022 (e, f), the two spilled oils collected in early 2022 (g, h), and the two spilled oils collected in 2019 (i, j).

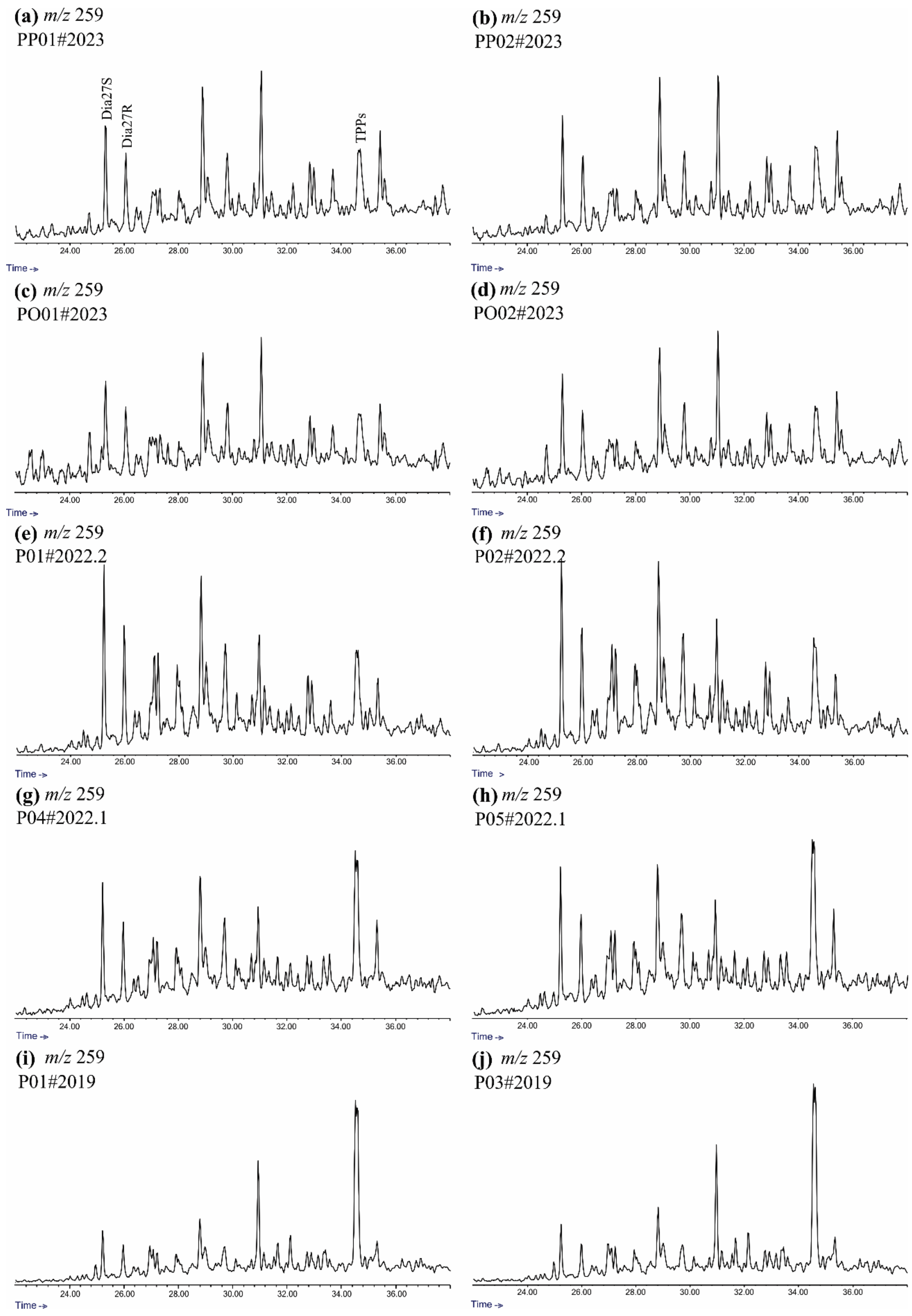

Figure S5. Selected ion chromatograms *m/z* 259, showing the C27 diasteranes (Dia27 S and R) and C30 tetracyclic polyprenoids (TPPs), of the four 2023 spilled oil at the State of Bahia (a-d), in addition to the two tarballs collected in late 2022 (e, f), the two spilled oils collected in early 2022 (g, h), and the two spilled oils collected in 2019 (i, j).

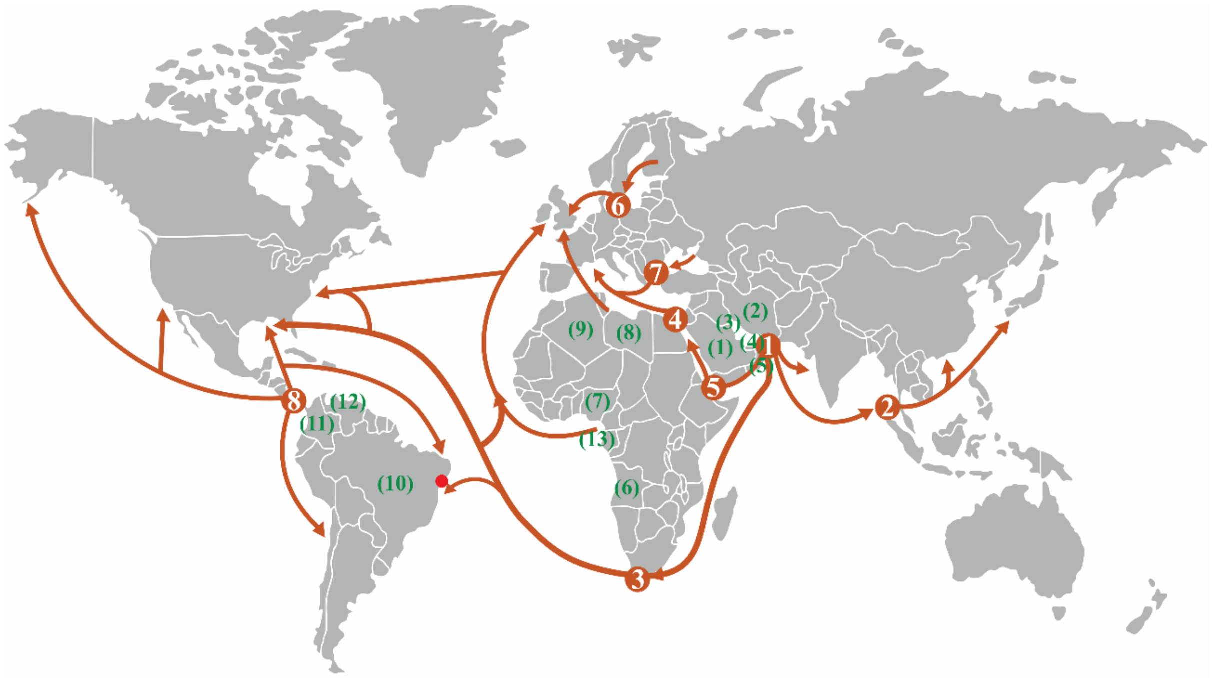

● Salvador, Bahia: area affected by the 2023 spilled oil

Crude oil exporter countries:

(1) Saudi Arabia (2) Iran (3) Kuwait (4) United Arab Emirates (5) Oman (6) Angola (7) Nigeria (8) Libya (9) Algeria (10) Brazil (11) Colombia (12) Venezuela (13) Gabon

Oil Passages:

1 Stralt of Hormuz 2 Strait of Malacca 3 Cape of Good Hope 4 Suez Canal 5 Bab-el-Mandeb 6 Danish Straits 7 Turkish Straits 8 Panama Canal

Figure S6. Map showing the country's exporters of crude oil considered herein,[15,16] the maritime transportation routes of crude oil, and some critical oil passages.[17] The location of the 2023 spilled oil samples collection at Salvador city, State of Bahia, Northeast of Brazil, is also indicated (red dot).

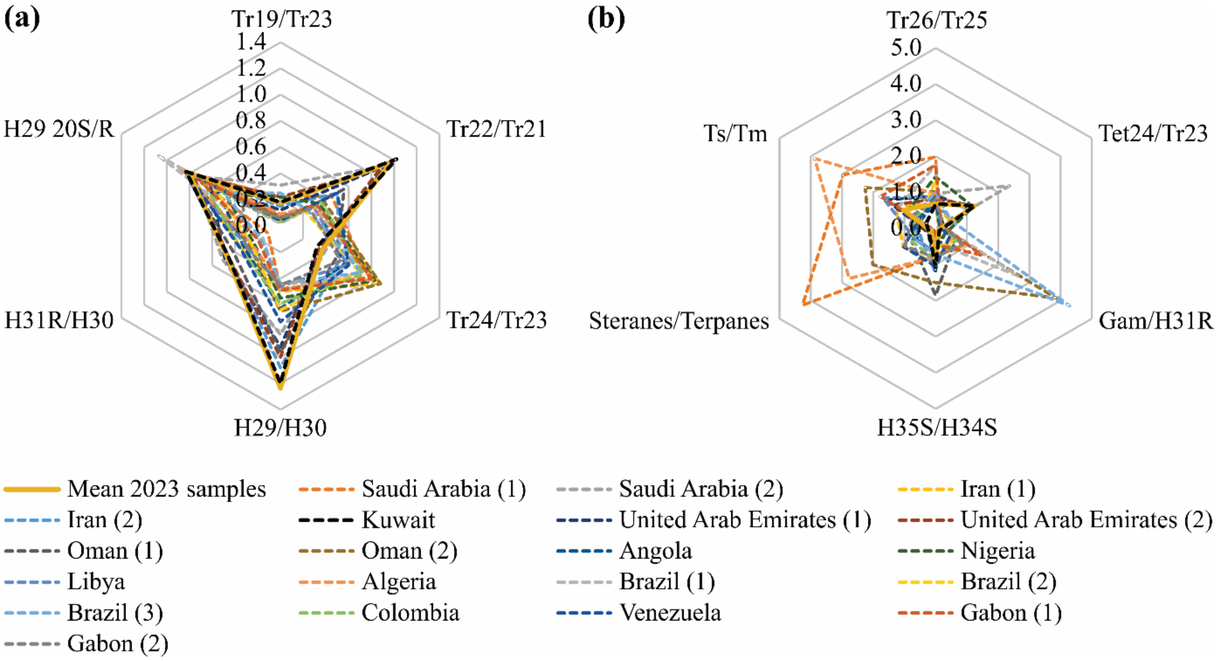

Figure S7. Radar plots comparing the diagnostic ratios based on the terpanes and steranes among the 2023 oil spill (average for the four samples) and oils produced in crude oil exporter countries from the Middle East (Saudi Arabia, Iran, Kuwait, United Arab Emirates, and Oman), Africa (Angola, Nigeria, Libya, Algeria, and Gabon) and South America (Brazil, Colombia, and Venezuela) (a and b; data from Peters et al.[12]).

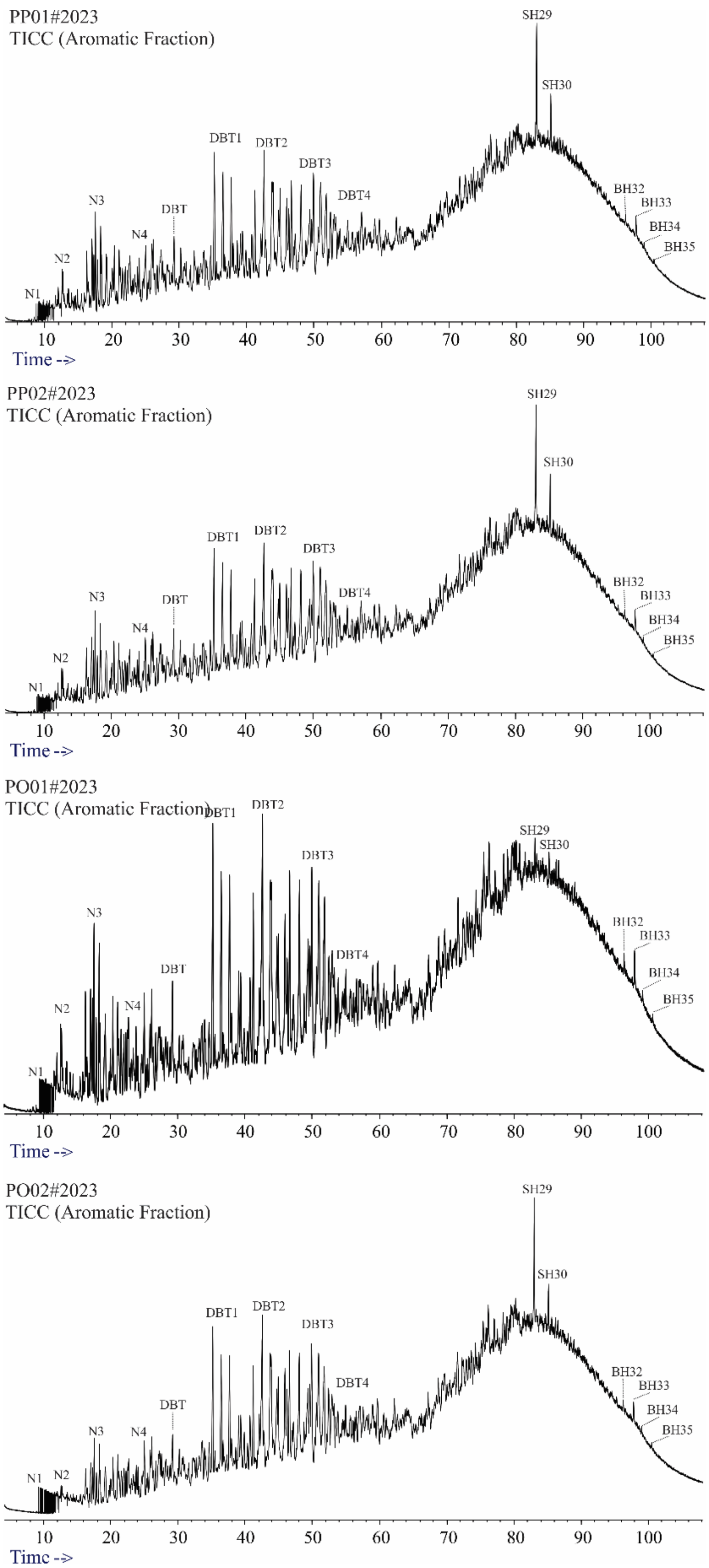

Figure S8. Total ion current chromatograms (TICC) for the aromatic fraction of the four 2023 spilled oil at the State of Bahia, showing the high abundance of akyldibenzothiophenes, aromatic 8,14- 8,14-secohopanes, and benzohopanes. Alkylphenanthrenes are also present. Ph1: methyl phenanthrene; Ph2: methyl phenanthrene; Ph3: trimethyl phenanthrene; DBT: dibenzothiophene; DBT1: methyl dibenzothiophenes; DBT2: dimethyl dibenzothiophenes; DBT3: trimethyl dibenzothiophenes; DBT4: tetramethyl dibenzothiophenes; SH29: aromatic C29 8,14-secohopane; SH30: C30 monoaromatic 8,14-secohopane; BH32: C32 benzohopane; BH33: C33 benzohopane; BH34: C34 benzohopane; BH35: C35 benzohopane.

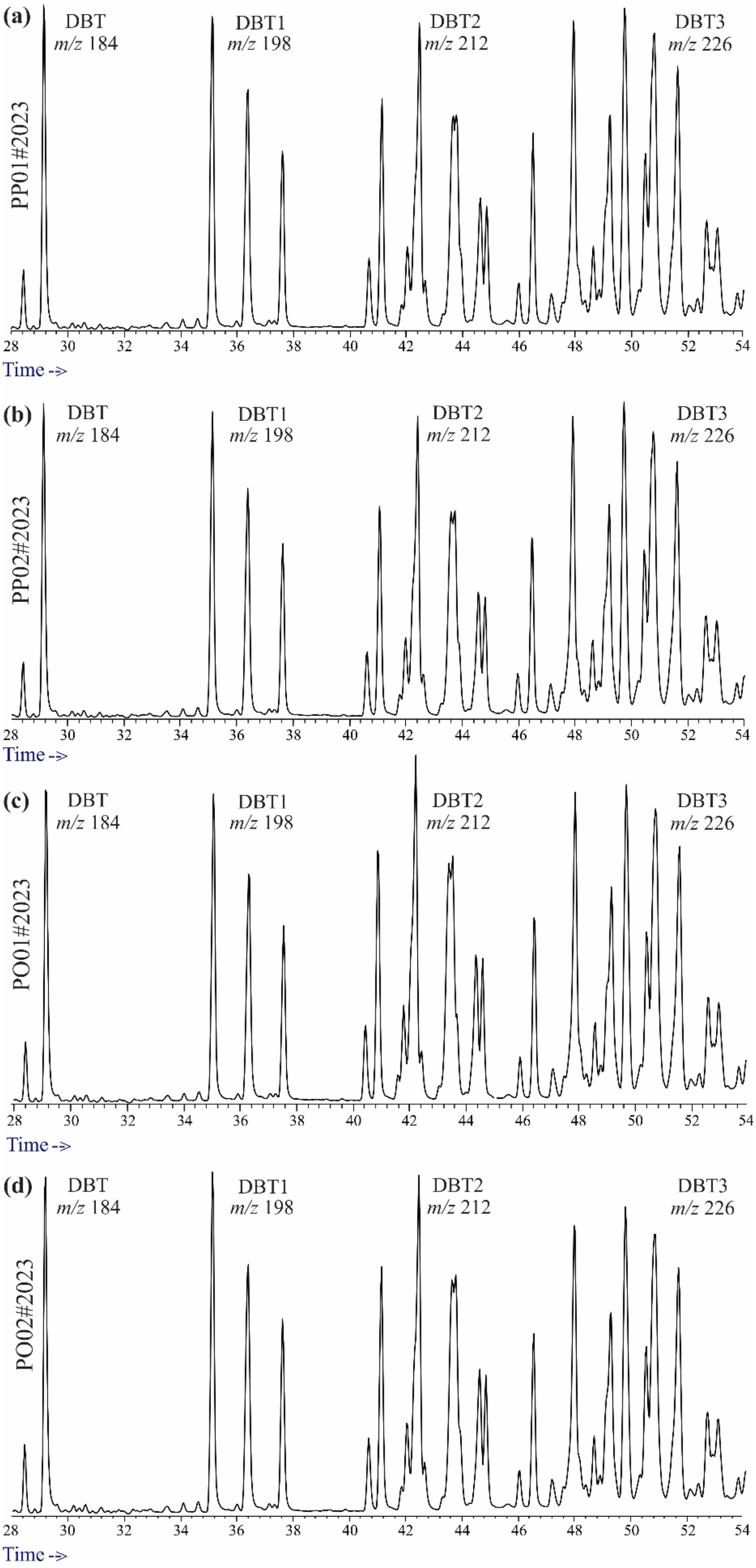

Figure S9. Selected ion chromatograms *m/z* 184, 198, 212, and 226 of the four 2023 spilled oil at the State of Bahia, presenting the distribution of the dibenzothiophene (DBT) and their alkylated series (DBT1, DBT2, and DBT3).

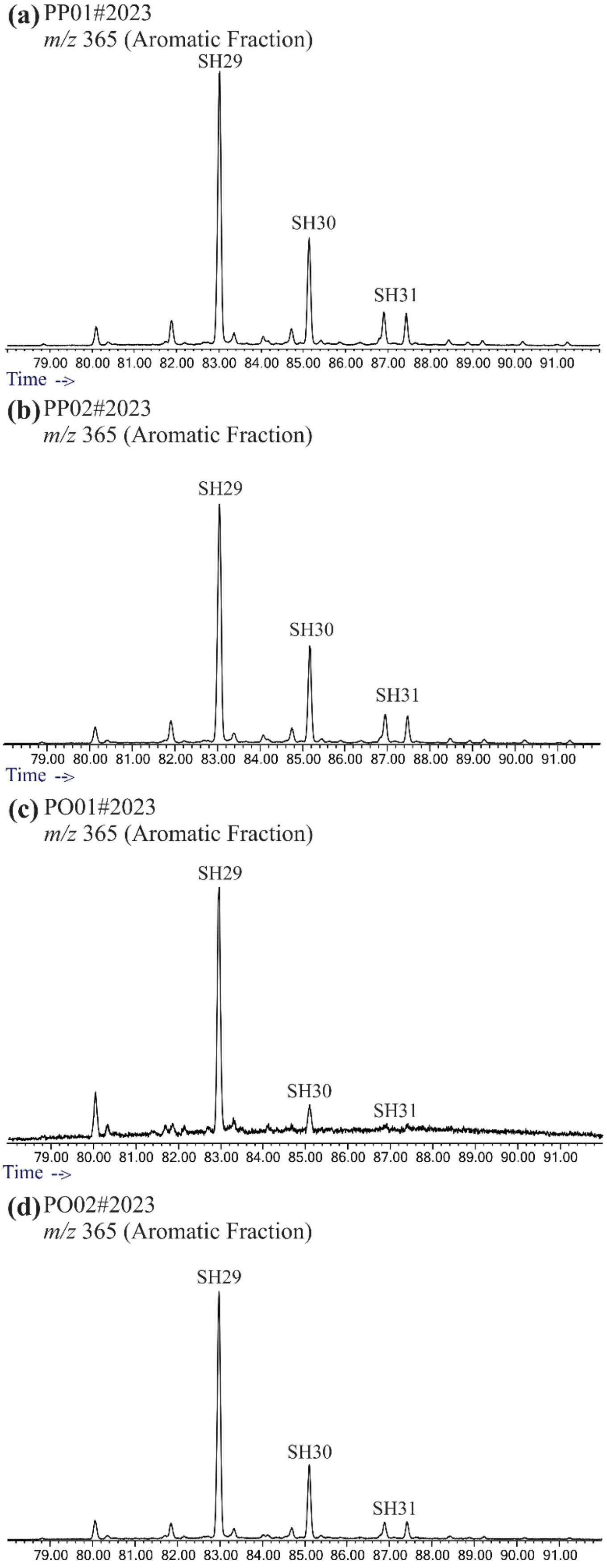

Figure S10. Selected ion chromatogram *m/z* 365 of the four 2023 spilled oil at the State of Bahia (a-d), presenting the distribution of the monoaromatic 8,14-secohopanes. SH29: aromatic C29 8,14-secohopane; SH30: C30 monoaromatic 8,14-secohopane; SH31: aromatic C31 8,14-secohopane.

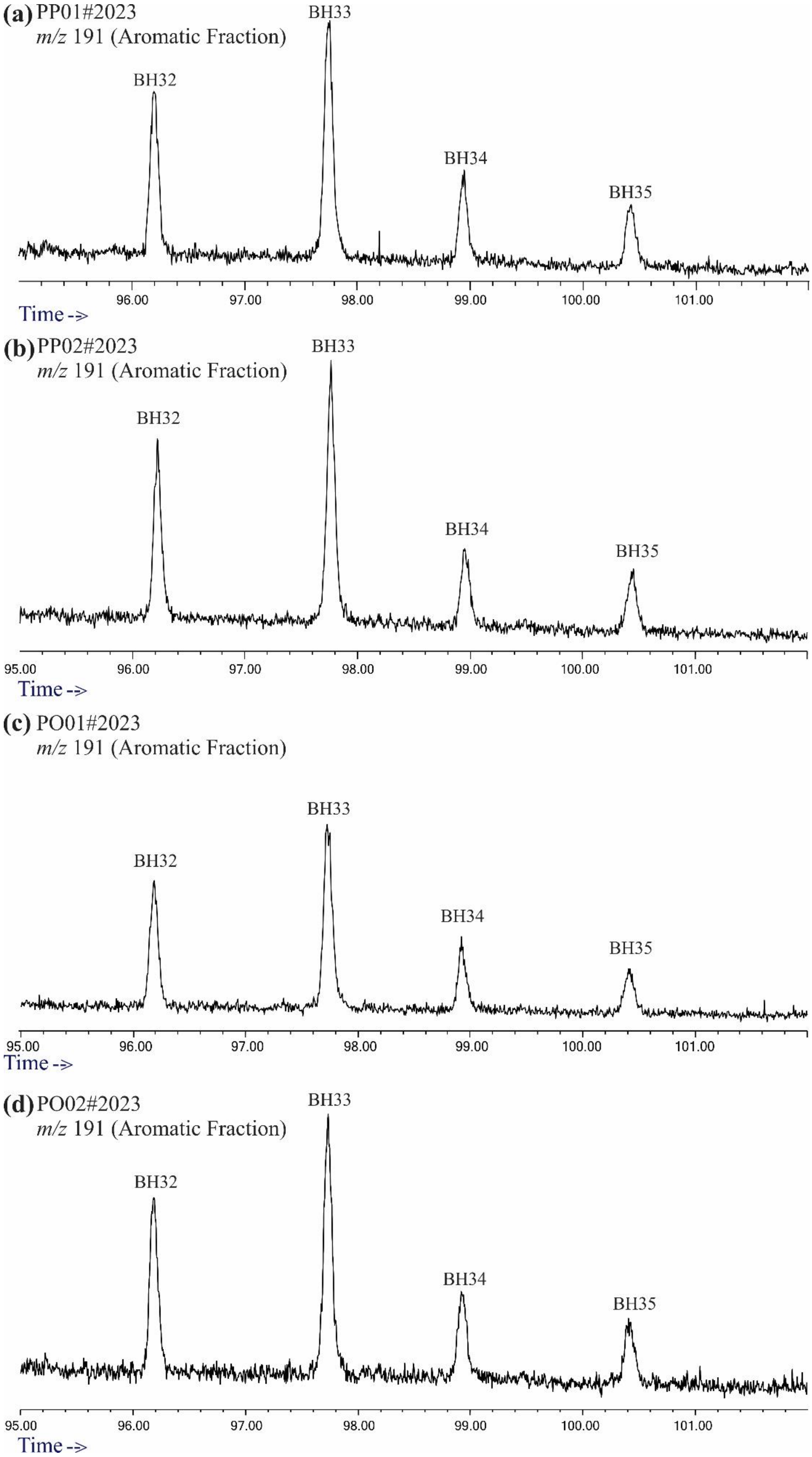

Figure S11. Selected ion chromatogram *m/z* 191 of the four 2023 spilled oil at the State of Bahia, presenting the distribution of the C32-C35 regular benzohopanes. BH32: C32 benzohopane; BH33: C33 benzohopane; BH34: C34 benzohopane; BH35: C35 benzohopane.

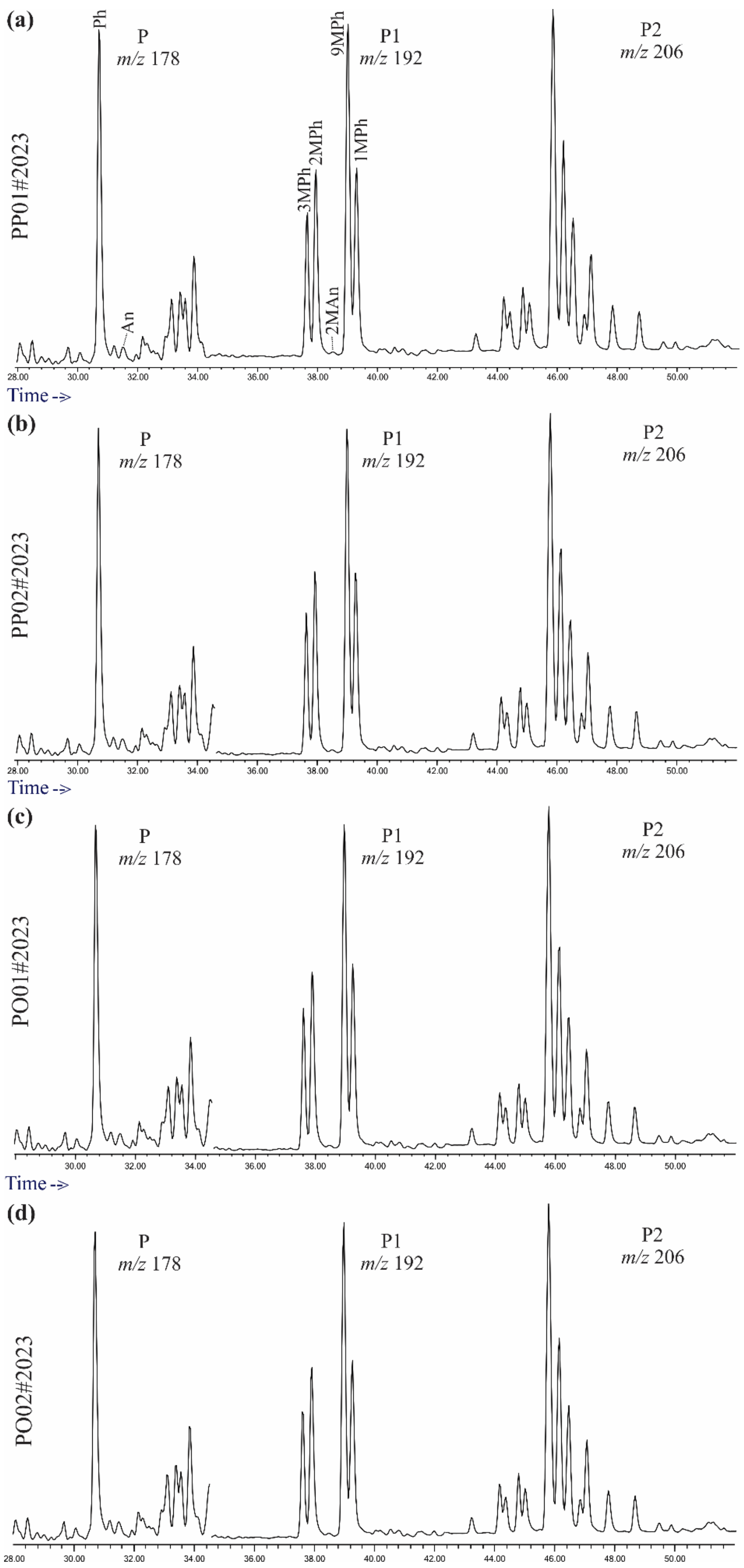

Figure S12. Selected ion chromatograms *m/z* 178, 192, and 206 of the four 2023 spilled oil at the State of Bahia, presenting the distribution of the phenanthrene (P) and their alkylated series (P1 and P2). In addition, it is tentatively identified the anthracene (An) and 2-methyl-anthracene (2MAn).

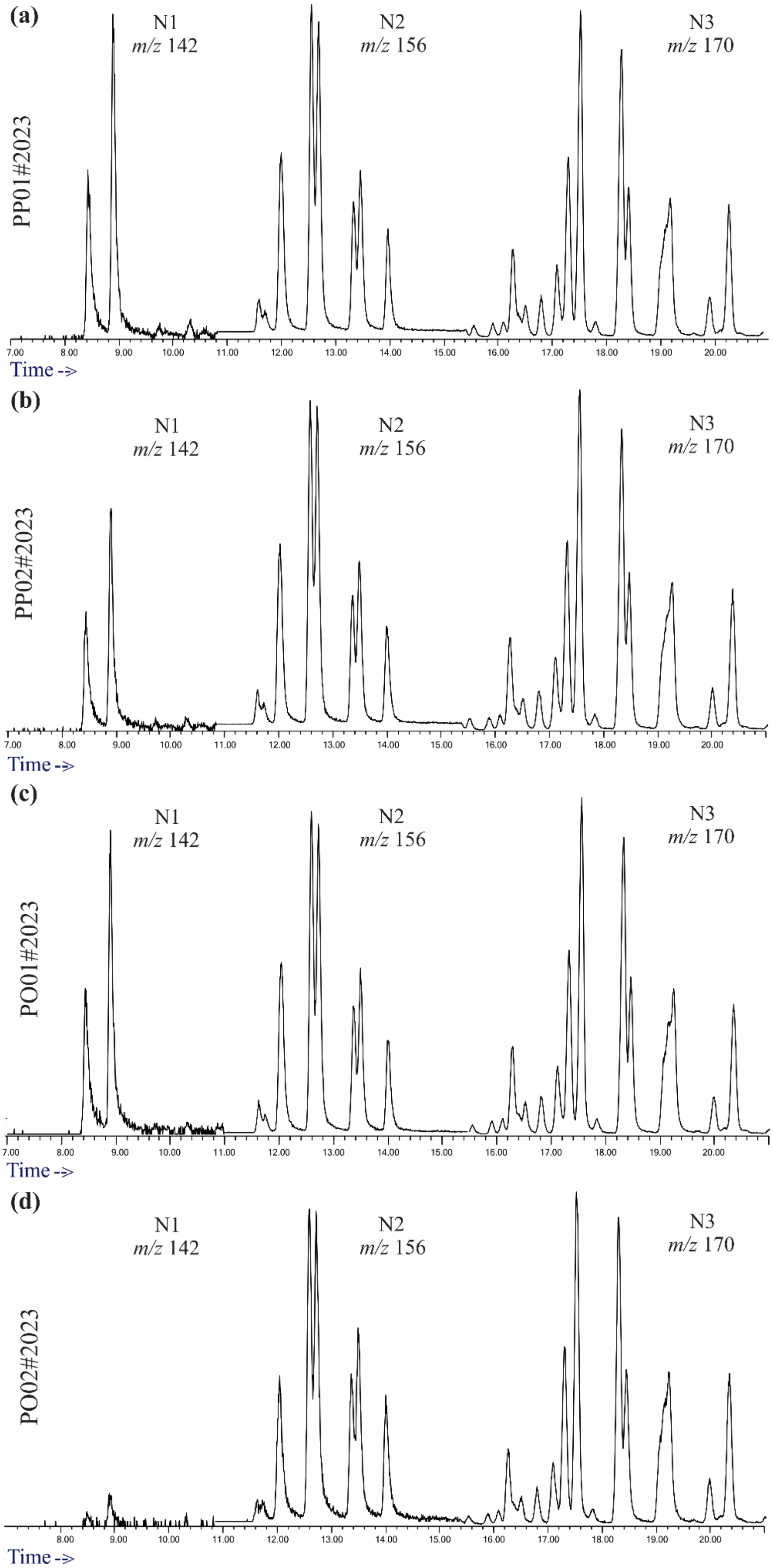

Figure S13. Selected ion chromatograms *m/z* 142, 156, and 170 of the four 2023 spilled oil at the State of Bahia, presenting the distribution of the alkylated naphthalene's series (N1, N2, and N3).

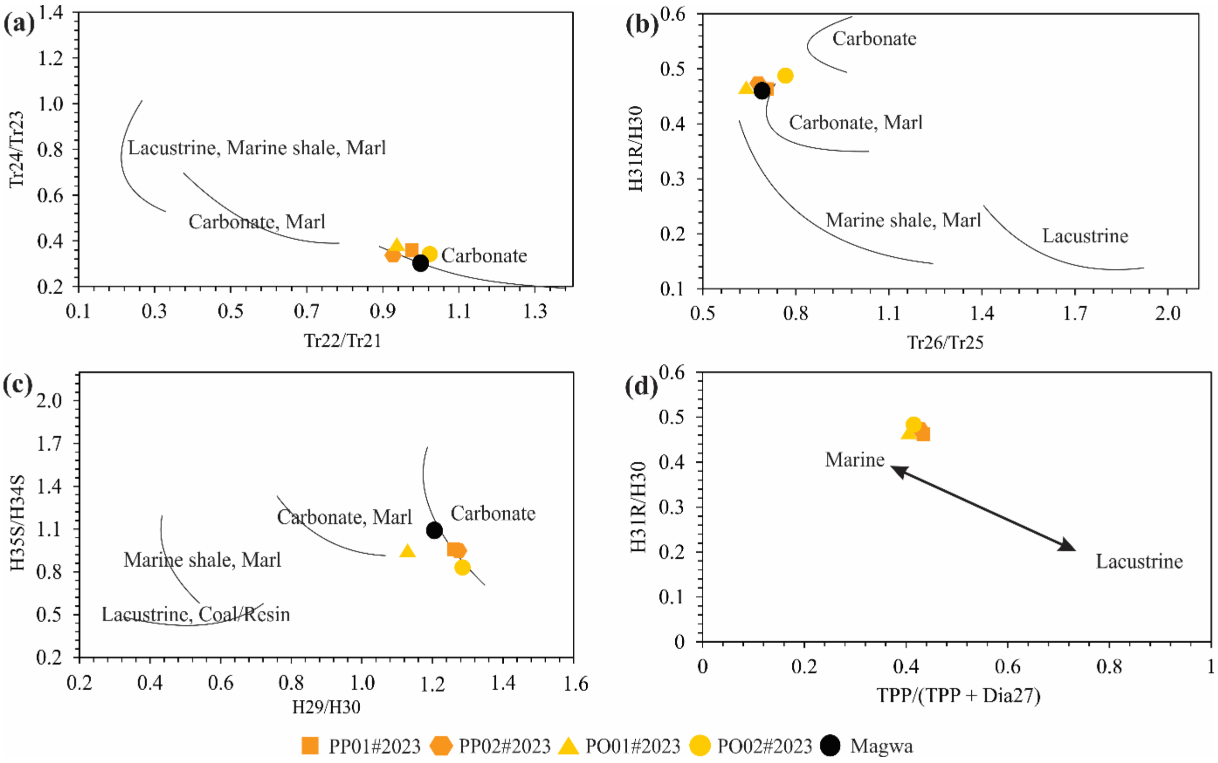

Figure S14. Plots to assess the source rock and depositional environment of the four 2023 spilled oil samples:[12,18,19] (a) Tr22/Tr21 *vs.* Tr24/Tr23; (b) Tr26/Tr25 *vs.* H31R/H30; (c) H29/H30 *vs.* H35S/H34S; (d) TPP/(TPP + Dia27) *vs.* H31R/H30. The oil from Magwa field was added in graphics a, b, c according to the ratios values provided by Peters et al.[12]

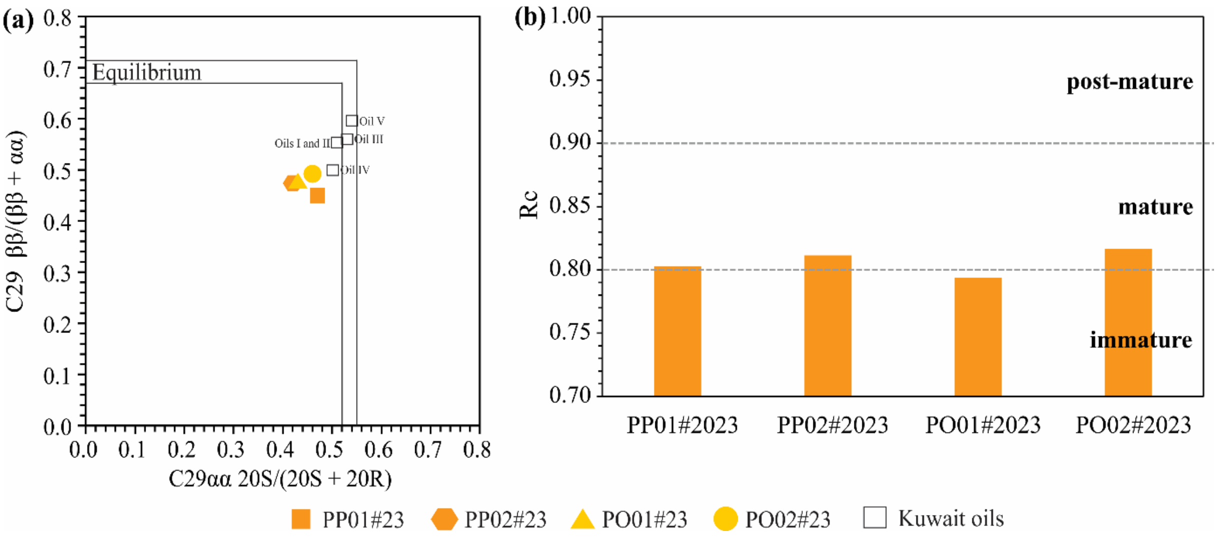

Figure S15. Plots to assess thermal maturity of the four 2023 spilled oil samples: (a) C29 20S/20 (S + R) *vs.* C29 ββ/C29 (ββ + αα); (b) Bar graphic showing the values of the calculated vitrinite reflectance (Rc) based on the methylphenanthrene index (MPI-1; Radke[20]). Rc = 0.60MPI-1 + 0.40.

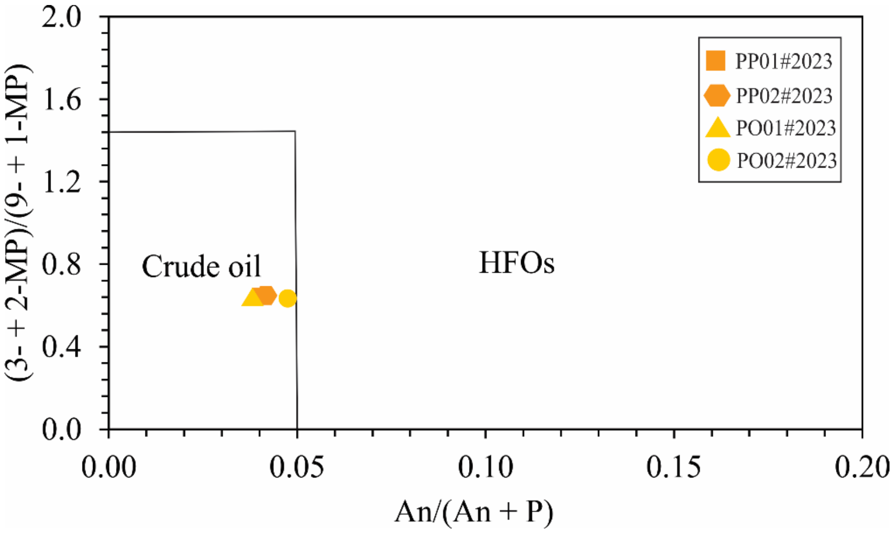

Figure S16. Plot to assess type of oil for the four 2023 spilled oil samples: Anthracene/(Anthracene + Phenanthrene) *vs.* (3- + 2- methylphenanthrene)/(9/4- + 1-methylphenanthrene).

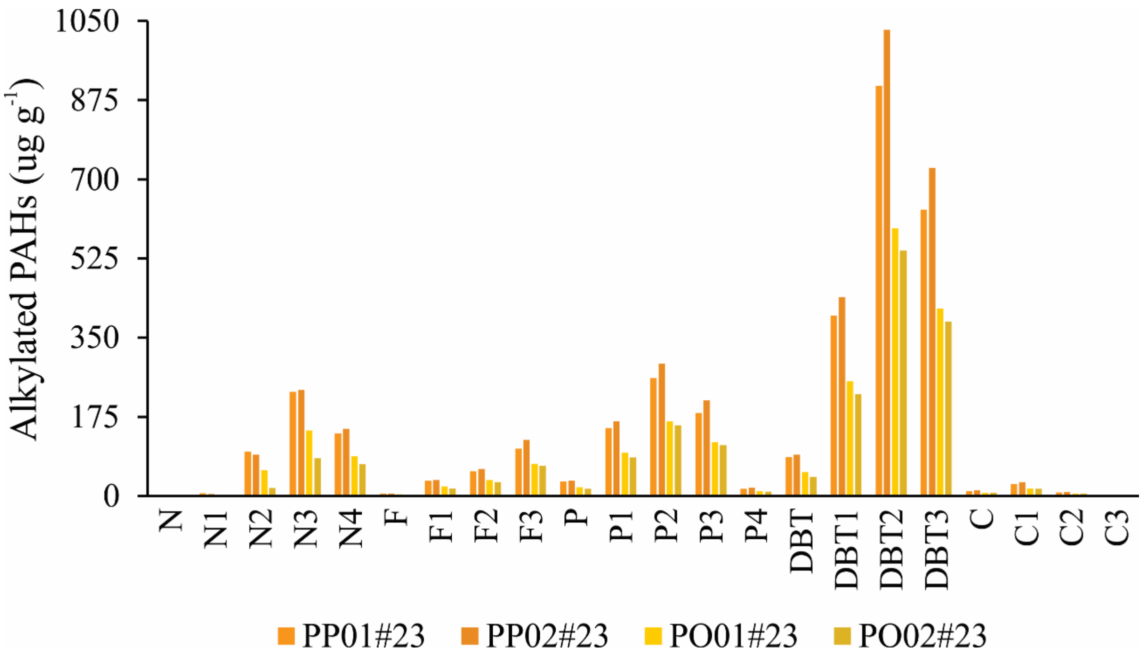

Figure S17. Profile of PAHs for the four 2023 spilled oil samples, presenting the distribution of them and their alkyl homologues: naphthalenes (N, N1, N2, and N3), fluorenes (F, F1, F2, and F3), phenanthrenes (P, P1, P2, P3, and P4), dibenzothiophenes (DBT, DBT1, DBT2, DBT3), and chrysenes (C, C1, C2, and C3).

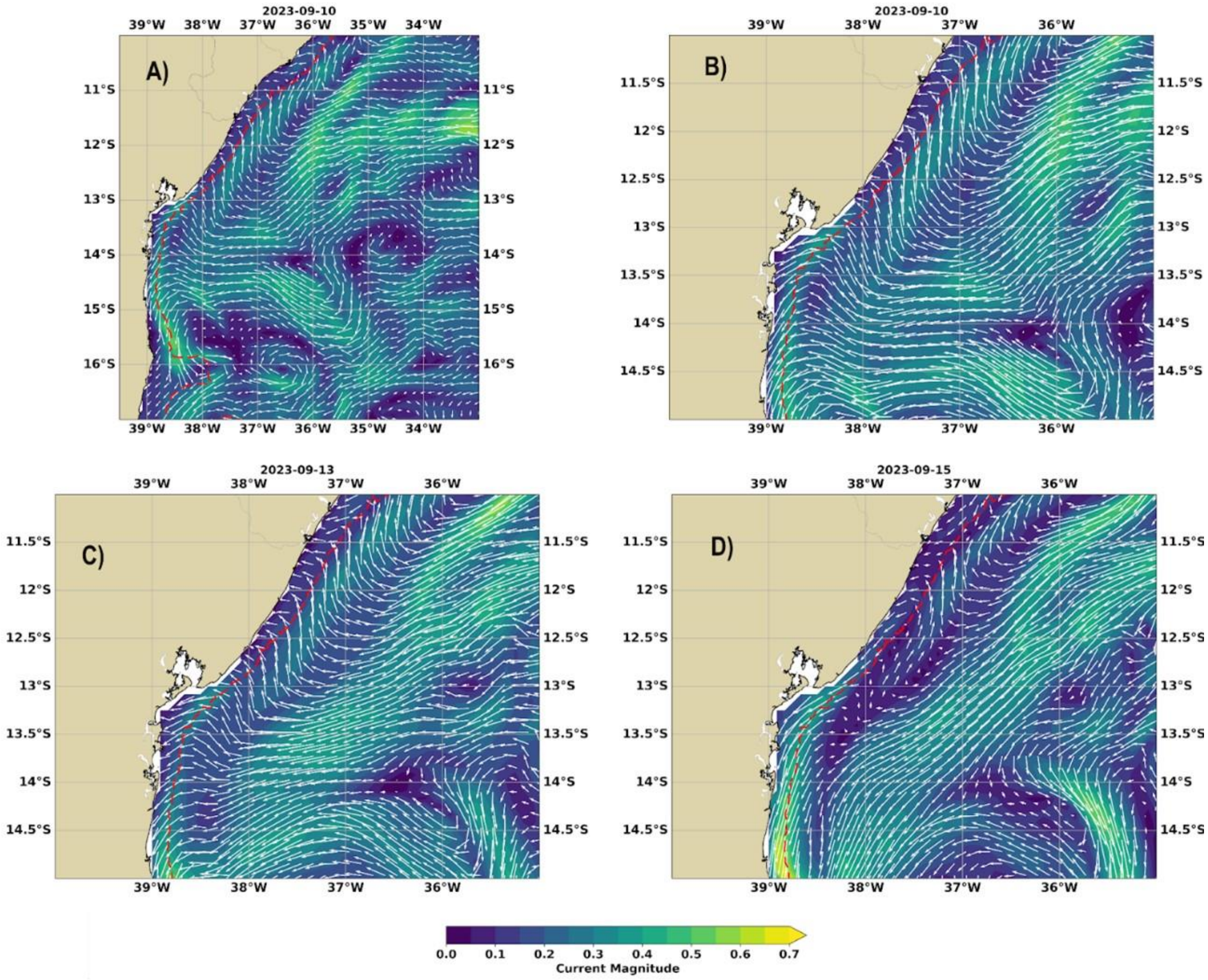

Figure S18. Daily-mean GLORYS-MERCATOR ocean surface circulation in the tropical southwestern Atlantic Ocean between September 10th and 15th, 2023: (a) overview of the Tropical Atlantic, showing the two eddies present in this region during the study period, (b) detailed view of the region adjacent to where the oil has arrived on September 10th and (c) 13th, and (d) closeup of the coastal area affected by the oil spill on September 15th. The red dots indicate the locations from which beached oil was reported. The dashed red line represents the 1000 m isobath.

References

[1] G1, 2023a. Manchas de óleo são encontradas em pelo menos seis praias de Salvador. https://g1.globo.com/ba/bahia/noticia/2023/09/10/manchas-de-oleo-sao-encontradas-em-praias-de-salvador.ghtml. (Assessed 12 November 2023).

[2] Governo do Estado da Bahia, 2023. Reunião define ações para combater ressurgimento de manchas de óleo em praias baianas. https://www.meioambiente.ba.gov.br/noticia/2024-02/14446/reuniao-define-acoes-para-combater-ressurgimento-de-manchas-de-oleo-em-praias. (Assessed 12 November 2023).

[3] G1, 2023b. Manchas de óleo aparecem em praia no baixo sul da Bahia . https://g1.globo.com/ba/bahia/noticia/2023/09/13/manchas-de-oleo-aparecem-em-praia-na-ba.ghtml. (Assessed 12 November 2023).

[4] UFBA, 2023. Análises do LEPETRO UFBA indicam correlação entre óleo derramado em Salvador e petróleo do Kuwait. Assessed. https://www.ufba.br/ufba_em_pauta/analises-do-lepetro-ufba-indicam-correlacao-entre-oleo-derramado-em-salvador-e. (Accessed 12 November 2022).

[5] IBAMA, 2023. Ibama coleta fragmentos de óleo no litoral da Bahia. https://www.gov.br/ibama/pt-br/assuntos/noticias/2023/ibama-coleta-fragmentos-de-oleo-no-litoral-da-bahia. (Assessed 12 November 2023).

[6] Carregosa, J. C.; Santos, I. R. D.; De Sá, M. S.; Santos, J.M.; Wisniewski, A. Multiple reaction monitoring tool applied in the geochemical investigation of a mysterious oil spill in northeast Brazil. *An. Acad. Bras. Cienc.* **2021**, 93, 1–20. https://doi.org/10.1590/0001-3765202120210171

[7] Terra, W. S.; Martins, L. L.; da Cruz, G. F. Avaliação da Eficiência de Diferentes Solventes Orgânicos na Precipitação de Asfaltenos de Petróleos Brasileiros e Análise das Frações Asfaltênicas e Maltênicas por Diferentes Técnicas Instrumentais. *Rev. Virtual Quim.* **2019**, 11, 1344-1363. https://doi.org/10.21577/1984-6835.20190093

[8] Martins, L. L.; Schulz, H.-M.; Noah, M.; Potz, S.; Ribeiro, H. J. P. S.; Cruz, G. F. New paleoenvironmental proxies for the Irati black shales (Paraná Basin, Brazil) based on acidic NSO compounds revealed by ultra-high resolution mass spectrometry. *Org. Geochem.* **2021**, 151, 104152. https://doi.org/10.1016/j.orggeochem.2020.104152

[9] Azevedo, D. A.; Silva, T. F.; Silva, D. B. Avaliação geoquímica de biomarcadores ocluídos em estruturas asfaltênicas. *Quim. Nova* **2009**, 32, 7, 1770-1776. https://doi.org/10.1590/S0100-40422009000700017

[10] Marotta, E.; Aquino, F. R.; Azevedo, D. A. Separação e determinação quantitativa dos alcanos lineares e dos cíclicos/ramificados em petróleos brasileiros por aduto de ureia e cromatografia gasosa: um estudo de caso revisitado. *Quím. Nova* **2014**, 37, 10, 1692–1698. https://doi.org/10.5935/0100-4042.20140265

[11] Lima, B. D.; Martins, L. L.; Pereira, V. B.; Franco, D. M. M.; Santos, I.R.; Santos, J. M.; Vaz, B. G.; Azevedo, D. A.; Cruz, G. F. Weathering impacts on petroleum biomarker, aromatic, and polar compounds in the spilled oil at the northeast coast of Brazil over time. *Mar. Pollut. Bull.* **2023**, 189, 114744. https://doi.org/10.1016/j.marpolbul.2023.114744

[12] Peters, K. E.; Walters, C. C.; Moldowan, J. M. The Biomarker Guide: Biomarkers and Isotopes in the Petroleum Exploration and Earth History, 2nd ed., University Press: Cambridge, **2005**, vol. 2.

[13] Abdullaha, F. H.; Connan, J. Geochemical study of some Cretaceous rocks from Kuwait: comparison with oils from Cretaceous and Jurassic reservoirs. *Org. Geochem.* **2002**, 33, 125–148. https://doi.org/10.1016/S0146-6380(01)00143-7

[14] Hauser, A.; Dashtib, H.; Khanb, Z. H. Identification of biomarker compounds in selected Kuwait crude oils. *Fuel* **1999**, 78, 1483-1488. https://doi.org/10.1016/S0016-2361(99)00075-7

[15] Wang, S.; Jia, H.; Lu, J.; Yang, D. Crude oil transportation route choice: A connectivity reliability-based approach. *Reliab. Eng. Syst. Saf.* **2023**, 235, 109254. https://doi.org/10.1016/j.ress.2023.109254

[16] Ostic, D.; Twum, A. K.; Agyemang, A. O.; Boahen, H.A. Assessing the impact of oil and gas trading, foreign direct investment inflows, and economic growth on carbon emission for OPEC member countries. *Environ. Sci. Pollut. Res.* **2022**, 29, 43089-43101. https://doi.org/10.1007/s11356-021-18156-0

[17] Salimi, M.; Amidpour, M. The Impact of Energy Transition on the Geopolitical Importance of Oil-Exporting Countries. *World* **2022**, 3, 607-618. https://doi.org/10.3390/world3030033

[18] Bastos, L. P. H.; Cavalcante, D. C.; Alferes, C. L. F.; Silva, D. B. N.; Ferreira, L. O.; Rodrigues, R.; Pereira, E. Fingerprinting an oil spill event (August of 2021) in the oceanic Fernando de Noronha archipelago using biomarkers and stable carbon isotopes. *Mar. Pollut. Bull.* **2022**, 185, 114316. https://doi.org/10.1016/j.marpolbul.2022.114316

[19] Holba, A. G.; Tegelaar, E.; Ellis, L.; Singletary, M. S.; Albrecht, P. Tetracyclic polyprenoids: indicators of freshwater (lacustrine) algal input. *Geology* **2000**, 28, 251–254. https://doi.org/10.1130/0091-7613(2000)28<251:TPIOFL>2.0.CO;2

[20] Radke, M. Application of aromatic compounds as maturity indicators in source rocks and crude oils. *Mar. Pet. Geol.* **1988**, 5, 224-236. https://doi.org/10.1016/0264-8172(88)90003-7
